# Association between levels of physical activity and low handgrip strength: Korea National Health and Nutrition Examination Survey 2014-2019

**DOI:** 10.4178/epih.e2022027

**Published:** 2022-02-21

**Authors:** Hyungsoon Ahn, Hwa Young Choi, Moran Ki

**Affiliations:** Department of Cancer Control and Population Health, Graduate School of Cancer Science and Policy, National Cancer Center, Goyang, Korea

**Keywords:** Sarcopenia, Hand strength, Physical activity, Korean adults

## Abstract

**OBJECTIVES:**

This study aimed to investigate the association between levels of physical activity (PA) and low handgrip strength in Korean adults.

**METHODS:**

Our cross-sectional study design included 24,109 Korean adults older than 19 years of age who participated in the Korea National Health and Nutrition Examination Survey 2014–2019. Low handgrip strength is described as hand strength less than the cut-off value of the 20th percentile of handgrip strength from a healthy population in each gender and age group. PA was categorized into three levels (inactive, active, and highly active) according to the World Health Organization’s global recommendations on PA for health. Multivariable logistic regression analysis was used to examine the association between levels of PA and low handgrip strength.

**RESULTS:**

Odds ratios (ORs) for low handgrip strength were significantly higher in middle-aged women who were active (adjusted odds ratio [aOR], 1.40; 95% confidence interval [CI], 1.15 to 1.69) and inactive (aOR, 1.47; 95% CI, 1.23 to 1.76) than in those highly active in walking exercise. Most of older people had significantly higher ORs for low handgrip strength in active compared to highly active in the context of aerobic, muscle strengthening, and walking exercise.

**CONCLUSIONS:**

Walking exercise was associated with a lower risk of sarcopenia in middle-aged women and older individuals. However, further studies are necessary to confirm the causal relationship between levels of PA and low handgrip strength.

## INTRODUCTION

Sarcopenia is a progressive disease related to falls, fractures, physical disabilities, and death. It is a musculoskeletal disorder generally accompanied by the reduction of musculoskeletal mass and muscular strength [1]. Sarcopenia, previously recognized as a natural consequence of aging, has been redefined as a pathophysiological condition, especially after it was assigned a disease code in 2016 International Classification of Diseases, 10th revision, Clinical Modification [2]. The criteria for sarcopenia diagnosis were regularly updated based on scientific evidence. In 2010, the European Working Group on Sarcopenia in Older People (EWGSOP1) preferentially recommended using reduction of muscle mass as a measure for diagnosis of sarcopenia. However, based on the scientific evidence that muscular strength is a better predictor of adverse sarcopenia outcomes than muscle mass, the EWGSOP2 updated their recommendation to using the reduction of muscular strength for diagnosis of sarcopenia in 2018 [1,3]. The EWGSOP2 more systematically established sarcopenia diagnostic criteria as follows: identification of potential sarcopenia based on a decrease in muscular strength, confirmation of sarcopenia based on low muscle quantity or low muscle quality, and estimation of sarcopenia severity based on the additional measurement of low physical performance [1]. In addition to the new diagnostic criteria, the EWGSOP2 emphasized that sarcopenia could exist in younger age groups [1], which highlights that muscle mass and muscular strength should be maintained from a younger age to prevent or delay sarcopenia. Thus, if secondary factors of sarcopenia including disease, undernutrition, and lack of physical activity (PA), are addressed in younger generations, later and long-term manifestations of sarcopenia in older age groups could be prevented and delayed. According to the new sarcopenia diagnosis algorithm of the EWGSOP2, handgrip strength can be utilized for diagnosing sarcopenia and evaluating the decline of muscular strength, in which it is sufficient to initiate intervention to improve sarcopenia for those who show handgrip strength below the reference levels (27 kg for men and 16 kg for women) in clinical practice [1].

PA is an important intervention method for sarcopenia [4-7]. Resistance exercise is a representative PA related to the prevention and treatment of sarcopenia; upper body exercises focused on muscular strength enhancement of the upper limbs have positive effects on improving handgrip strength [8]. Moreover, a combination of balance, flexibility, and endurance exercises, along with resistance exercises, could be helpful in handgrip strength improvement [9]. However, most studies focused on the relationship between PA and handgrip strength in the older population specifically; thus, sarcopenia in young people is poorly understood. Lee et al. [10] observed that handgrip strength begins to decline in middle age, regardless of gender, based on a study population consisting of those 10 years or older in Korea. Similarly, Kim et al. [11] demonstrated that average handgrip strength decreases in Koreans of both genders in their 40s. Since both studies were cross-sectional study, their results cannot determine the effect of fourth decade handgrip strength loss on the development of sarcopenia in old age. However, both studies recommend PA during middle age, when the loss of handgrip strength begins, for protection against sarcopenia. Dodds et al. [12] also showed that sarcopenia in old age could be prevented based on muscle accumulated previously, such as from middle age. As such, being physically active during middle age is recommended.

The relationship between PA and low handgrip strength in older Koreans has been well-studied; however, no study has analyzed this relationship in the Korean population aged 19 years or older [13]. By targeting healthy Koreans who participated in the Korea National Health and Nutrition Examination Survey (KNHANES) 2014-2019 aged ≥ 19 years, we investigated the reference values of low handgrip strength, subdivided by gender and age group, and analyzed the relationship between low handgrip strength and the levels of aerobic, muscle strengthening, and walking exercises.

## MATERIALS AND METHODS

### Data collection and study participants

Of 45,022 participants in the 2014-2019 KNHANES, we selected 33,687 individuals aged 19 years or older whose handgrip strength on the dominant hand was measured 3 times. Finally, 24,109 individuals who responded to the survey on height, weight, body mass index (BMI), education level, household income, smoking status, frequency of alcohol drinking, comorbidities (hypertension, diabetes, musculoskeletal disease, hypercholesterolemia, hypertriglyceridemia), and PAs (aerobic, muscle strengthening, and walking exercise) were included in the study.

### Handgrip strength and cut-off values

Since 2014, the KNHANES has been testing the handgrip strength of those who are 10 years or older using a digital grip strength dynamometer (Takei Scientific Instruments Co., Ltd, Tokyo, Japan). This study used the maximum handgrip strength values from three measurements of the dominant hand and six measurements in cases of ambidextrous individuals. Cut-off values of low handgrip strength were defined as the bottom 20th percentile of handgrip strength values after dividing the healthy population depending on gender and age group (19-29, 30-39, 40-49, 50-59, 60-69, and ≥ 70 years) [14]. Of those whose handgrip strength on the dominant hand was measured 3 times, the healthy population was first selected from those who answered “no” to the question, “Do you have any limitations in daily and social activities due to health issues or physical or mental disorders?” among the questions related to inactivity and quality of life in the KNHANES; then answered, “no problems” to the five questions (mobility, self-care, usual activities, pain/discomfort, and anxiety/depression) of EuroQol 5 dimension (EQ-5D) that measures health-related quality of life (n= 21,462) [15]. For men, the final cut-off values of low handgrip strength were 35.7 kg for 19-29 years, 38.2 kg for 30-39 years, 37.4 kg for 40-49 years, 36.1 kg for 50-59, 33.0 kg for 60-69 years, and 27.3 kg for ≥ 70 years. For women, the cut-off values were 20.6 kg for 19-29 years, 22 kg for 30-39 years, 22 kg for 40-49 years, 21.3 kg for 50-59 years, 19.8 kg for 60-69 years, and 16.1 kg for ≥ 70 years.

### Physical activity

PA was assessed based on the self-reported responses to Global Physical Activity Questionnaire provided to those aged ≥ 19 years in KNHANES, in which PA types were categorized into aerobic, walking, and muscle strengthening exercises. Aerobic exercise was evaluated based on the metabolic equivalent task (MET) that considered exercise intensity using PA time (minutes) related to work, transport, and leisure. According to the PA guidelines of the World Health Organization (WHO), work and leisure-related PAs were evaluated through the calculation of weekly MET (MET-min/wk) by multiplying 8 MET for high-intensity PAs and 4 MET for moderate-intensity PAs [16]. Transport-related PAs were calculated by multiplying 4 MET for moderate-intensity to obtain weekly MET (MET-min/wk). Walking exercise also conformed to the WHO guidelines, in which PA time (minutes) was multiplied by 4 MET to obtain weekly MET (MET-min/wk) of PA [16]. Referring to the PA guidelines of the WHO, individuals with < 600 MET minutes, 600≤ MET minutes< 1,200, and ≥ 1,200 MET minutes of aerobic and walking exercise per week were classified into inactive, active, and highly active groups, respectively. Moreover, those with < 2 days, 2-3 days, and ≥ 4 days of muscle strengthening exercise per week were divided into inactive, active, and highly active groups, respectively.

### Covariates

Based on preceding studies, the socio-demographic factors affecting low handgrip strength were BMI (underweight, BMI< 18.5; normal, 18.5 ≤ BMI < 23.0; overweight, 23.0 ≤ BMI < 25.0; and obese, 25.0≤ BMI), education level (≤ elementary school, middle school, high school, and ≥ college), and household income (lowest, mid-low, mid-high, and highest). Health behavior-related factors used were smoking status (never, past, and currently smoking) and frequency of alcohol consumption (never, < 1, 1-4, and ≥ 5 times/mo) [11,17-23]. The considered comorbidities were hypertension, diabetes, musculoskeletal disease, hypercholesterolemia, and hypertriglyceridemia. The reference values of hypertension were 140 mmHg or higher in systolic blood pressure and 90 mmHg or higher in diastolic blood pressure, and those who answered “yes” to the question about taking an antihypertensive drug were considered hypertensive. Patients with 126 mg/dL or higher fasting blood sugar and those who were receiving insulin injections, taking an antidiabetic drug, or were diagnosed with diabetes by a doctor were considered to have diabetes. The criteria for those with musculoskeletal disease were to have arthritis, osteoarthritis, rheumatoid arthritis, or osteoporosis. The criteria for those with hypercholesterolemia were a total cholesterol level > 240 mg/dL or cholesterol-lowering medication use. Individuals with hypertriglyceridemia were defined as those with > 200 mg/dL fasting triglyceride level at 12 hours.

### Statistical analysis

All statistical analyses were performed using weights generated by stratified multi-stage clustered sampling. To calculate p-values for categorical variables, chi-square test and one-way analysis of variance were applied, while p-values for continuous variables were obtained using the t-test. To analyze the relationship between PA level and low handgrip strength, multivariable logistic regression analysis was used to calculate the prevalence odds ratio (OR) of low handgrip strength for groups with either active or inactive PA levels compared to highly active PA levels as references. Model 1 was adjusted for age, gender, and BMI. Model 2 was adjusted for education level, household income, smoking status, and frequency of alcohol drinking and in addition to those of model 1. Model 3 was additionally adjusted for comorbidities (hypertension, diabetes, musculoskeletal disease, hypercholesterolemia, and hypertriglyceridemia) in addition to those of model 2. In the analysis of the relationship between aerobic and walking exercises and low handgrip strength, muscle strengthening exercise was adjusted as a covariate to more accurately analyze the relationship between PA level and low handgrip strength. The final analysis results are presented for the crude model without adjustment and for the adjusted model corresponding to the model 3, i.e., the results obtained after adjustment for all covariates. Complex sample design, as recommended by the KNHANES guidelines, was reflected in all analyses. SAS version 9.4 (SAS Institute Inc., Cary, NC, USA) was used for the analyses, in which the statistical significance level was p-value < 0.05 (Supplementary Materials 1-4).

### Ethics statement

This study was approved by the Institutional Review Board of the Korea Disease Control and Prevention Agency (IRB No. 2013- 12EXP-03-5C, 2018-01-03-P-A, 2018-01-03-C-A). This study conforms to the principles of the Declaration of Helsinki. All participants were provided with the informed consent form.

## RESULTS

The characteristics of the 24,109 participants are shown in Table 1. There were more women (51.2%) than men (48.8%). In all PAs, the inactive group accounted for majority of the study population. Among the participants performing muscle strengthening exercise, 77.5% were in the inactive group (less than 2 times/wk). Among those performing aerobic and walking exercise, there were more participants in the highly active group than in the active group.

Factors related to PA levels are summarized in Table 2. Overall, low handgrip strength most observed in the inactive group. The mean handgrip strength increased as the PA level increased. However, the post-hoc test found no significant difference in the mean handgrip strength between the inactive and active groups practicing walking exercise. Most men belonged to the highly active groups in all PAs, whereas women were mostly inactive, except in the context of walking exercise. In muscle strengthening exercise, 60s or older group mostly belonged to the highly active group (all p< 0.05).

Prevalence ORs showed that active and inactive groups had lower handgrip strength than highly active groups in terms of PA levels in the multivariable logistic regression analysis (Table 3). In the adjusted model analysis for all participants, active and inactive groups for aerobic exercise had ORs (adjusted odds ratio [aOR], 1.33; 95% CI, 1.18 to 1.49 and aOR, 1.37; 95% CI, 1.25 to 1.51, respectively) higher than that of the highly active group indicating significant increase in the OR of low handgrip strength. The inactive group for muscle strengthening and walking exercises had higher ORs (aOR, 1.68; 95% CI, 1.46 to 1.93 and aOR, 1.16; 95% CI: 1.06 to1.27, respectively) than the highly active group showing a significant increase in the OR of low handgrip strength. The active group for walking exercise showed a significant increase in the OR of low handgrip strength (aOR, 1.19; 95% CI, 1.08 to 1.30) compared to the highly active group.

In adjusted model analyses by gender of all participants, women gender in the active and inactive groups for walking exercise had a significant increase in OR of low handgrip strength (aOR, 1.24; 95% CI, 1.09 to 1.41 and aOR, 1.36; 95% CI, 1.21 to 1.52, respectively) compared to those in the highly active group. In contrast, men in the highly active group for walking exercise showed no significant differences in the OR of low handgrip strength from those in the active and inactive groups.

In adjusted model analyses of all participants by age group (19-39, 40-59, and ≥ 60 years), young participants (19-39 years) in the inactive group for muscle strengthening exercise showed higher OR (aOR, 1.93; 95% CI, 1.46 to 2.57) of low handgrip strength than those in the highly active group. Middle-aged participants (40-59 years) in the active and inactive groups for aerobic exercise showed a significant increase in the OR of low handgrip strength (aOR, 1.40; 95% CI, 1.17 to 1.67 and aOR, 1.54; 95% CI, 1.32 to 1.79, respectively) compared to those in the highly active group. The active group for walking exercise also showed a significant increase in OR compared to highly active group (aOR, 1.27; 95% CI, 1.09 to 1.48). Older individuals (aged ≥ 60 years) in inactive groups for aerobic, muscle strengthening, and walking exercises had a significant increase in OR of low handgrip strength (aOR, 1.33; 95% CI, 1.12 to1.59; aOR, 1.91; 95% CI, 1.53 to 2.37; and aOR, 1.25; 95% CI, 1.09 to 1.44), compared to the highly active group. Although the young, middle-aged, and older participants in the active groups for muscle strengthening exercise had higher ORs of low handgrip strength than those in the highly active group, there were no significant differences.

**Figure 1.**
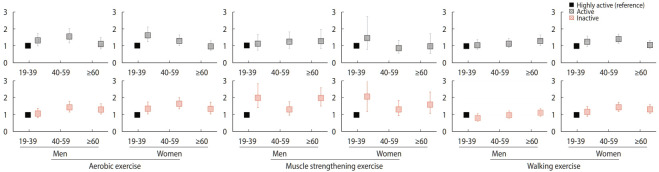
Adjusted odds ratios for low handgrip strength according to level of physical activity by gernder and age. Values are presented as odds ratio with 95% confidence interval.

Figure 1 shows the ORs of low handgrip strength by gender, age group, and PA in a subgroup analysis of all participants. For women in the young population, unlike the young men, the inactive group for aerobic exercise had a higher OR (aOR, 1.36; 95% CI, 1.05 to 1.75) for low handgrip strength than those in the highly active group. In the case of the middle-aged women, active and inactive groups for walking exercise showed a significant increase in the OR of low handgrip strength (aOR, 1.40; 95% CI, 1.15 to 1.69 and aOR, 1.47; 95% CI, 1.23 to 1.76, respectively) compared to those in the highly active group. As for the older women, the inactive group for each PA had a significantly higher OR of low handgrip strength than the highly active group. In contrast, the older men in the inactive groups for aerobic and muscle strengthening exercises had a significantly higher OR of low handgrip strength than those in the highly active group; those in the active group for walking exercise also had a significant increase (aOR, 1.30; 95% CI, 1.03 to 1.65) compared to those in the highly active group.

## DISCUSSION

Using the data of KNHANES 2014-2019 representing the Korean characteristics, the present study analyzed the relationship between PA level and low handgrip strength in Korean aged ≥ 19 years.

When the entire study participants were divided by gender, women engaged in walking exercise showed a significant relationship with low handgrip strength while men showed no relationship. Particularly, middle-aged women had a significant increase in the OR of low handgrip strength as the walking exercise level decreased. Considering that low handgrip strength is a preferential indicator for sarcopenia diagnosis according to the sarcopenia diagnosis guidelines of EWGSOP2, these findings suggest that walking exercise should have effects on the prevention of sarcopenia in Korean women [1]. Few studies have investigated the relationship of walking in middle-aged women with muscular strength and muscle mass. In a study on the effect of walking on body composition changes in middle-aged to older women aged ≥ 50 years, Gába et al. [24] compared the body composition between the brisk walking intervention (n= 58) and control (n= 46) group after 10 weeks of intervention and found was no significant difference between the groups. When body composition was compared before and after 10 weeks within the brisk walking intervention group, lean body mass of the lower limbs (kg) significantly increased, while that of the upper limbs (kg) showed no significant difference. In contrast to Gába et al. [24]’s study results, our study found a significant relationship between walking and handgrip strength, which is a test factor for upper limbs muscular strength. This difference is probably due to the following reasons: Gába et al. targeted middle-aged to older women who worked in offices mostly in seating positions, and applied the intervention only for 10 weeks; however, in the present study the participants comprised individuals from the general population in whom the relationship between walking and handgrip strength was investigated during daily activities. Moreover, the present study utilized the self-reported questionnaire for evaluating walking exercise levels, while Gába et al. directly measured step numbers to identify walking exercise levels. Men and women showed different relationships between walking exercise and handgrip strength; this may be attributable to the difference in body composition between men and women. Since men have more upper limb muscle mass than women, men might be found to gain more benefit from aerobic exercise or muscle strengthening exercise in terms of handgrip strength than from walking exercise [25].

The OR for handgrip strength in young participants in the inactive group for muscle strengthening exercise, regardless of gender, was approximately 2 times lower than that in those in the highly active group of the same exercise, indicating a relevant relationship between muscle strengthening exercise and low handgrip strength in the young population. However, no significant relationship between walking exercise and low handgrip strength was found in this population. This study indicated that handgrip strength increased during the second to third decade of life, and then started to decrease during the fourth (Supplementary Material 5). This suggests that muscle strengthening exercises could help prevent sarcopenia in the young population in their 20s to 30s, producing a high-level muscular strength.

In the present study, middle-aged individuals in active and inactive groups for aerobic exercise had a significantly higher OR of low handgrip strength than those in the highly active groups. Using the data of the KNHANES 2014-2017, Seong et al. [26] studied Koreans aged ≥ 19 years, in which the group inactive for aerobic exercise (active: ≥ 600 MET-min/wk, and inactive: < 600 MET-min/wk) had a significantly higher OR of low handgrip strength than the active group. These results are similar to those of our study. Thus, aerobic exercise has the potential to prevent sarcopenia in middle-aged individuals.

Walking exercise affected the OR of low handgrip strength in older individuals. In the case of older women, the inactive group showed a more significant increase in the OR of low handgrip strength than the highly active group, whereas older men in the active group had a significantly higher OR of low handgrip strength than those in the highly active group. Thus, walking exercise could reduce sarcopenia risk in older individuals of both genders. This, however, should be carefully interpreted, considering that older individuals with active muscle strengthening exercise could be active in both aerobic and walking exercises. Older individuals in muscle strengthening and walking exercise tended to be more engaged in the highly active group than those in the active group. Considering that the 2020 National Survey on Sports Participation found lack of time as the primary reason for not engaging in PA, it can be speculated that older individuals could spare more time for muscle strengthening or walking exercise because they have relatively more time than younger or middle-aged individuals [27]. Moreover, media emphasized that muscle strengthening exercises prevent chronic diseases, such as osteoporosis and dementia, and walking exercise exerts a lower load on the body in older individuals. These may be relevant to the high activity noted in the two exercise types [28,29].

This study has some limitations. First, it was a cross-sectional study; therefore, we were unable to identify a causal relationship. A prospective cohort study should be conducted to investigate the causal relationship between PA level for each age group and low handgrip strength. Second, PAs were divided into aerobic, muscle strengthening, and walking exercises, and the participants were divided into highly active, active, and inactive groups for each PA. However, there is no clear distinction in each PA. This study only investigated the effect of PA level on low handgrip strength; thus, the exclusion of participants overlapping between PAs would have substantially decreased the sample size, compromising the data quality representing Koreans. In the future, an additional study should be performed considering the cross effects of PAs or with clear distinction among PAs. Third, information on PA level was collected based on a self-reported questionnaire; therefore, the relationship between PA level and low handgrip strength might have been inaccurately estimated as a result of bias. Finally, as this study was based on the KNHANES data targeting Koreans, it has limitations in generalization to other countries. Nonetheless, this study has provided information about the relationship between low handgrip strength and PA level in Korean adults, which can be used as fundamental data to establish a sarcopenia prevention strategy targeting both older and younger populations.

## Figures and Tables

**Table 1.** General characteristics of the study participants, Korea National Health and Nutrition Examination Survey 2014-2019

Characteristics	Unweighted, n	Weighted, n (%)
Total	24,109	27,422,844
Gender		
Men	10,407	13,389,066 (48.8)
Women	13,702	14,033,778 (51.2)
Age (yr)		
19-39	6,582	9,842,704 (35.9)
40-59	8,971	10,975,543 (40.0)
≥60	8,556	6,604,596 (24.1)
BMI (kg/m^2^)		
Underweight (<18.5)	930	1,168,874 (4.3)
Normal (18.5-23.0)	9,411	10,797,840 (39.4)
Overweight (23.0-25.0)	5,595	6,204,454 (22.6)
Obesity (≥25.0)	8,173	9,251,675 (33.7)
Education level		
≤Elementary school	4,953	3,929,267 (14.3)
Middle school	2,432	2,330,891 (8.5)
High school	7,854	9,837,841 (35.9)
≥College	8,870	11,324,844 (41.3)
Household income		
Lowest	4,505	4,122,288 (15.0)
Mid-low	5,993	6,622,083 (24.1)
Mid-high	6,653	8,125,632 (29.6)
Highest	6,958	8,552,841 (31.2)
Smoking status		
Never	14,790	16,051,334 (58.5)
Past	5,249	5,872,520 (21.4)
Current	4,070	5,498,990 (20.1)
Frequency of alcohol drinking (times/mo)		
Never	2,696	2,452,568 (8.9)
<1	8,479	9,047,340 (33.0)
1-4	7,851	9,834,276 (35.9)
≥5	5,083	6,088,660 (22.2)
Comorbidity^1^		
Hypertension	7,665	7,240,361 (26.4)
Diabetes	3,001	2,781,893 (10.1)
Musculoskeletal Disease	3,632	2,961,530 (10.8)
Hypercholesterolemia	5,328	5,278,101 (19.2)
Hypertriglyceridemia	3,452	4,068,663 (14.8)
Low handgrip strength		
Yes	5,376	5,891,853 (21.5)
No	18,733	21,530,990 (78.5)
Aerobic exercise (MET-min/wk)		
Inactive (<600)	13,154	14,030,545 (51.2)
Active (600-1,200)	4,721	5,474,614 (20.0)
Highly active (≥1,200)	6,234	7,917,684 (28.9)
Muscle strengthening exercise (day/wk)		
Inactive (<2)	19,083	21,264,487 (77.5)
Active (2-3)	2,547	84,537 (11.8)
Highly active (≥4)	2,479	77,444 (10.6)
Walking exercise (MET-min/wk)		
Inactive (<600)	9,744	10,791,966 (39.4)
Active (600-1,200)	6,144	7,206,408 (26.3)
Highly active (≥1,200)	8,221	9,424,469 (34.4)

BMI, body mass index; MET, metabolic equivalent task.

1 Comorbidity displays populations who have the disease.

**Table 2.** Characteristics of study participants according to levels of physical activity, Korea National Health and Nutrition Examination Survey 2014-2019

Variables	Aerobic exercise			p-value^1^	Muscle strengthening exercise			p-value^1^	Walking exercise			p-value^1^
	Inactive	Active	Highly active		Inactive	Active	Highly active		Inactive	Active	Highly active	
Unweighted, n	13,154	4,721	6,234		19,083	2,547	2,479		9,744	6,144	8,221	
Weighted, n	14,030,545	5,474,614	7,917,684		21,264,487	3,246,034	2,912,323		10,791,966	7,206,408	9,424,469	
Handgrip strength (kg)	30.76±0.12	31.67±0.18	35.08±0.17	<0.001	31.03±0.10	35.75±0.24	36.68±0.25	<0.001	31.98±0.15^2^	31.95±0.17^2^	32.60±0.14	0.001
Low handgrip strength												
Yes	3,295 (24.1)	1,038 (21.9)	1,043 (16.5)	<0.001	4,576 (23.1)	423 (16.5)	377 (15.3)	<0.001	2,405 (23.3)	1,397 (21.9)	1,574 (19.1)	<0.001
No	9,859 (75.9)	3,683 (78.1)	5,191 (83.5)		14,507 (76.9)	2,124 (83.5)	2,102 (84.7)		7,339 (76.7)	4,747 (78.1)	6,647 (80.9)	
Gender												
Men	5,267 (45.0)	1,927 (45.8)	3,213 (57.6)	<0.001	7,332 (43.8)	1,410 (61.2)	1,665 (71.9)	<0.001	4,187 (49.2)	2,525 (46.9)	3,695 (49.8)	0.011
Women	7,887 (55.0)	2,794 (54.2)	3,021 (42.4)		11,751 (56.2)	1,137 (38.8)	814 (28.1)		5,557 (50.8)	3,619 (53.1)	4,536 (50.2)	
Age (yr)												
19-39	2,884 (29.1)	1,439 (38.9)	2,259 (45.8)	<0.001	5,056 (34.6)	934 (45.2)	592 (35.0)	<0.001	2,464 (32.8)	1,914 (40.4)	2,204 (36.0)	<0.001
40-59	4,786 (40.9)	1,833 (40.6)	2,352 (38.0)		7,115 (40.5)	1,027 (40.2)	829 (36.4)		3,662 (42.0)	2,272 (38.2)	3,037 (39.2)	
≥60	5,484 (30.0)	1,449 (20.4)	1,623 (16.2)		6,912 (24.9)	586 (14.6)	1,058 (28.6)		3,618 (25.2)	1,958 (21.3)	2,980 (24.9)	

Data are expressed as the estimated mean±standard error for continuous variables and frequency (weighted %) for categorical variables.

1 Using t-test for continuous variables and chi-square test for categorical variables.

2 The means difference is not significant at the 0.05 level by Scheffe post-hoc test.

**Table 3.** Odds ratios for low handgrip strength according to levels of physical activity

Variables^1^			Aerobic exercise^2^			Muscle strengthening exercise			Walking exercise^2^		
			Highly active	Active	Inactive	Highly active	Active	Inactive	Highly active	Active	Inactive
Total											
	Crude		1.00 (reference)	1.42 (1.27, 1.59)	1.60 (1.46, 1.76)	1.00 (reference)	1.09 (0.92, 1.30)	1.66 (1.46, 1.90)	1.00 (reference)	1.19 (1.08, 1.30)	1.28 (1.18, 1.40)
	Adjusted		1.00 (reference)	1.33 (1.18, 1.49)	1.37 (1.25, 1.51)	1.00 (reference)	1.17 (0.98, 1.39)	1.68 (1.46, 1.93)	1.00 (reference)	1.19 (1.08, 1.30)	1.16 (1.06, 1.27)
Men^3^											
	Crude		1.00 (reference)	1.48 (1.26, 1.74)	1.49 (1.30, 1.70)	1.00 (reference)	1.17 (0.94, 1.46)	1.72 (1.45, 2.04)	1.00 (reference)	1.14 (0.99, 1.31)	1.10 (0.96, 1.25)
	Adjusted		1.00 (reference)	1.36 (1.15, 1.61)	1.27 (1.11, 1.46)	1.00 (reference)	1.25 (0.99, 1.57)	1.74 (1.46, 2.07)	1.00 (reference)	1.14 (0.99, 1.32)	0.99 (0.86, 1.13)
Women^3^											
	Crude		1.00 (reference)	1.40 (1.21, 1.63)	1.76 (1.55, 1.99)	1.00 (reference)	0.97 (0.73, 1.30)	1.62 (1.29, 2.03)	1.00 (reference)	1.24 (1.10, 1.41)	1.50 (1.34, 1.67)
	Adjusted		1.00 (reference)	1.32 (1.13, 1.55)	1.50 (1.31, 1.71)	1.00 (reference)	1.04 (0.78, 1.39)	1.58 (1.26, 1.99)	1.00 (reference)	1.24 (1.09, 1.41)	1.36 (1.21, 1.52)
Age (yr)											
	19-39										
		Crude	1.00 (reference)	1.52 (1.27, 1.83)	1.33 (1.13, 1.58)	1.00 (reference)	1.15 (0.83, 1.60)	1.74 (1.33, 2.27)	1.00 (reference)	1.15 (0.97, 1.36)	1.11 (0.93, 1.32)
		Adjusted	1.00 (reference)	1.43 (1.18, 1.73)	1.17 (0.97, 1.40)	1.00 (reference)	1.20 (0.86, 1.68)	1.93 (1.46, 2.57)	1.00 (reference)	1.13 (0.95, 1.34)	0.98 (0.82, 1.18)
	40-59										
		Crude	1.00 (reference)	1.45 (1.21, 1.73)	1.66 (1.44, 1.92)	1.00 (reference)	1.03 (0.78, 1.35)	1.33 (1.08, 1.65)	1.00 (reference)	1.25 (1.08, 1.45)	1.26 (1.10, 1.46)
		Adjusted	1.00 (reference)	1.40 (1.17, 1.67)	1.54 (1.32, 1.79)	1.00 (reference)	1.09 (0.83, 1.44)	1.35 (1.09, 1.67)	1.00 (reference)	1.27 (1.09, 1.48)	1.22 (1.05, 1.41)
	≥60										
		Crude	1.00 (reference)	1.17 (0.95, 1.43)	1.72 (1.46, 2.03)	1.00 (reference)	1.10 (0.79, 1.52)	2.18 (1.78, 2.68)	1.00 (reference)	1.18 (1.01, 1.38)	1.55 (1.35, 1.77)
		Adjusted	1.00 (reference)	1.03 (0.83, 1.26)	1.33 (1.12, 1.59)	1.00 (reference)	1.20 (0.86, 1.67)	1.91 (1.53, 2.37)	1.00 (reference)	1.15 (0.98, 1.35)	1.25 (1.09, 1.44)

Values are presented as odds ratio (95% confidence interval).

1 Adjusted: adjusted for age, gender, body mass index, education level, household income, smoking status, frequency of alcohol drinking, and comorbidities (hypertension, diabetes, musculoskeletal disease, hypercholesterolemia, and hypertriglyceridemia).

2 We adjusted for muscle strengthening exercise, which could influence the association between physical activity and handgrip strength.

3 Gender was not adjusted.

## SUPPLEMENTARY MATERIALS

Korean version is available at https://www.e-epih.org/.

Supplementary Material 1. Characteristics of study participants according to levels of physical activity, KNHANES 2014-2019

Supplementary Material 2. Multivariable logistic regression analysis on low handgrip strength associated with levels of physical activity

Supplementary Material 3. Multivariable logistic regression analysis on low handgrip strength associated with levels of physical activity in men

Supplementary Material 4. Multivariable logistic regression analysis on low handgrip strength associated with levels of physical activity in women

Supplementary Material 5. Mean handgrip strength by age groups in men and women.
