# Supplementary material for: Association between levels of physical activity and low handgrip strength: Korea National Health and Nutrition Examination Survey 2014-2019
Source: Epidemiol Health. 2022 Feb 21;44:e2022027. doi: 10.4178/epih.e2022027 (PMC9117110; doi:10.4178/epih.e2022027)
Supplement: Supplementary Material 1. — Characteristics of study participants according to levels of physical activity, KNHANES 2014-2019 [file epih-44-e2022027-suppl1.docx]

| **Supplementary Material 1. Characteristics of study participants according to levels of physical activity, KNHANES 2014-2019** | | | | | | | | | | | | |
| --- | --- | --- | --- | --- | --- | --- | --- | --- | --- | --- | --- | --- |
| **Variables** | **Aerobic exercise** | | | **P value** | **Muscle strengthening exercise** | | | **P value** | **Walking exercise** | | | **P value** |
|  | Inactive | Active | Highly active |  | Inactive | Active | Highly active |  | Inactive | Active | Highly active |  |
| Unweighted N | 13,154 | 4,721 | 6,234 |  | 19,083 | 2,547 | 2,479 |  | 9,744 | 6,144 | 8,221 |  |
| Weighted N | 14,030,545 | 5,474,614 | 7,917,684 |  | 21,264,487 | 3,246,034 | 2,912,323 |  | 10,791,966 | 7,206,408 | 9,424,469 |  |
| Handgrip strength (kg) | 30.8 ± 0.12 | 31.7 ± 0.18 | 35.1 ± 0.17 | <.001* | 31.0 ± 0.10 | 35.8 ± 0.24 | 36.7 ± 0.25 | <.001* | 32.0 ± 0.15† | 32.0 ± 0.17† | 32.6 ± 0.14 | 0.001* |
| Low handgrip strength |  |  |  |  |  |  |  |  |  |  |  |  |
| Yes | 3,295 (24.1) | 1,038 (21.9) | 1,043 (16.5) | <.001** | 4,576 (23.1) | 423 (16.5) | 377 (15.3) | <.001** | 2,405 (23.3) | 1,397 (21.9) | 1,574 (19.1) | <.001** |
| No | 9,859 (75.9) | 3,683 (78.1) | 5,191 (83.5) |  | 14,507 (76.9) | 2,124 (83.5) | 2,102 (84.7) |  | 7,339 (76.7) | 4,747 (78.1) | 6,647 (80.9) |  |
| Sex |  |  |  |  |  |  |  |  |  |  |  |  |
| Men | 5,267 (45.0 | 1,927 (45.8) | 3,213 (57.6) | <.001** | 7,332 (43.8) | 1,410 (61.2) | 1,665 (71.9) | <.001** | 4,187 (49.2) | 2,525 (46.9) | 3,695 (49.8) | 0.011** |
| Women | 7,887 (55.0) | 2,794 (54.2) | 3,021 (42.4) |  | 11,751 (56.2) | 1,137 (38.8) | 814 (28.1) |  | 5,557 (50.8) | 3,619 (53.1) | 4,536 (50.2) |  |
| Age groups |  |  |  |  |  |  |  |  |  |  |  |  |
| 19-39 | 2,884 (29.1) | 1,439 (38.9) | 2,259 (45.8) | <.001** | 5,056 (34.6) | 934 (45.2) | 592 (35.0) | <.001** | 2,464 (32.8) | 1,914 (40.4) | 2,204 (36.0) | <.001** |
| 40-59 | 4,786 (40.9) | 1,833 (40.6) | 2,352 (38.0) |  | 7,115 (40.5) | 1,027 (40.2) | 829 (36.4) |  | 3,662 (42.0) | 2,272 (38.2) | 3,037 (39.2) |  |
| 60+ | 5,484 (30.0) | 1,449 (20.4) | 1,623 (16.2) |  | 6,912 (24.9) | 586 (14.6) | 1,058 (28.6) |  | 3,618 (25.2) | 1,958 (21.3) | 2,980 (24.9) |  |
| Height (cm) | 163.4 ± 0.11 | 164.6 ± 0.16 | 167.0 ± 0.14 | <.001* | 163.8 ± 0.09 | 167.6 ± 0.20 | 167.9 ± 0.21 | <.001* | 164.2 ± 0.13 | 164.9 ± 0.15 | 165.0 ± 0.12 | <.001* |
| Weight (kg) | 63.9 ± 0.14 | 64.5 ± 0.22 | 67.3 ± 0.21 | <.001* | 64.3 ± 0.12 | 67.1 ± 0.32 | 67.8 ± 0.29 | <.001* | 64.6 ± 0.16 | 65.1 ± 0.20 | 65.4 ± 0.17 | 0.008* |
| BMI (kg/m^2^) |  |  |  |  |  |  |  |  |  |  |  |  |
| Underweight (BMI<18.5) | 533 (4.6) | 185 (4.2) | 212 (3.8) | 0.010** | 782 (4.6) | 92 (3.8) | 56 (2.2) | <.001** | 403 (4.5) | 249 (4.3) | 278 (4.0) | 0.765** |
| Normal (18.5≤BMI<23.0) | 5,044 (39.1) | 1,958 (41.6) | 2,409 (38.2) |  | 7,401 (39.3) | 1,082 (41.4) | 928 (37.6) |  | 3,776 (39.4) | 2,439 (39.7) | 3,196 (39.1) |  |
| Overweight (23.0≤BMI<25.0) | 3,056 (22.5) | 1,080 (22.3) | 1,459 (23.0) |  | 4,331 (21.9) | 589 (23.0) | 675 (27.5) |  | 2,215 (22.2) | 1,432 (22.6) | 1,948 (23.1) |  |
| Obesity (25.0≤BMI) | 4,521 (33.8) | 1,498 (31.8) | 2,154 (35.0) |  | 6,569 (34.2) | 784 (31.8) | 820 (32.7) |  | 3,350 (33.8) | 2,024 (33.4) | 2,799 (33.9) |  |
| Education level |  |  |  |  |  |  |  |  |  |  |  |  |
| ≤Elementary school | 3,496 (19.6) | 762 (11.2) | 695 (7.2) | <.001** | 4,379 (16.3) | 214 (5.3) | 360 (10.0) | <.001** | 2,494 (17.9) | 1,020 (11.4) | 1,439 (12.5) | <.001** |
| Middle school | 1,487 (10.1) | 429 (7.4) | 516 (6.5) |  | 1,989 (9.0) | 160 (4.7) | 283 (9.0) |  | 1,003 (9.1) | 561 (7.5) | 868 (8.6) |  |
| High school | 3,917 (32.9) | 1,589 (36.9) | 2,348 (40.4) |  | 6,003 (34.8) | 937 (39.3) | 914 (40.0) |  | 2,899 (33.1) | 2,093 (37.2) | 2,862 (38.0) |  |
| ≥College | 4,254 (37.5) | 1,941 (44.4) | 2,675 (45.9) |  | 6,712 (39.9) | 1,236 (50.7) | 922 (41.1) |  | 3,348 (39.9) | 2,470 (43.9) | 3,052 (40.9) |  |
| Household income |  |  |  |  |  |  |  |  |  |  |  |  |
| Lowest | 2,957 (18.1) | 737 (13.1) | 811 (11.0) | <.001** | 3,864 (16.3) | 242 (8.3) | 399 (13.3) | <.001** | 2,126 (17.0) | 973 (13.0) | 1,406 (14.3) | <.001** |
| Mid-low | 3,385 (25.1) | 1,152 (23.8) | 1,456 (22.8) |  | 4,798 (24.5) | 570 (21.9) | 625 (24.0) |  | 2,421 (24.1) | 1,543 (24.5) | 2,029 (24.0) |  |
| Mid-high | 3,462 (28.9) | 1,355 (29.8) | 1,836 (30.9) |  | 5,215 (29.6) | 750 (30.1) | 688 (29.5) |  | 2,569 (28.9) | 1,770 (30.5) | 2,314 (29.8) |  |
| Highest | 3,350 (28.0) | 1,477 (33.3) | 2,131 (35.4) |  | 5,206 (29.6) | 985 (39.6) | 767 (33.3) |  | 2,628 (30.0) | 1,858 (31.9) | 2,472 (32.0) |  |
| Smoking status |  |  |  |  |  |  |  |  |  |  |  |  |
| Never | 8,175 (59.1) | 3,031 (61.8) | 3,584 (55.3) | <.001** | 12,208 (60.8) | 1,415 (54.0) | 1,167 (46.9) | <.001** | 5,827 (56.2) | 3,922 (60.9) | 5,041 (59.4) | <.001** |
| Past | 2,769 (20.8) | 963 (20.2) | 1,517 (23.4) |  | 3,662 (19.1) | 655 (24.6) | 932 (35.0) |  | 2,024 (21.0) | 1,251 (20.1) | 1,974 (22.9) |  |
| Current | 2,210 (20.1) | 727 (18.0) | 1,133 (21.3) |  | 3,213 (20.1) | 477 (21.4) | 380 (18.1) |  | 1,893 (22.8) | 971 (19.0) | 1,206 (17.7) |  |
| Frequency of alcohol drinking |  |  |  |  |  |  |  |  |  |  |  |  |
| Never | 1,710 (10.7) | 483 (8.1) | 503 (6.3) | <.001** | 2,350 (10.0) | 147 (4.8) | 199 (6.1) | <.001** | 1,231 (10.4) | 598 (7.5) | 867 (8.4) | <.001** |
| Less than 1 time per month | 4,959 (35.4) | 1,604 (32.6) | 1,916 (29.1) |  | 6,965 (34.4) | 747 (27.3) | 767 (29.3) |  | 3,528 (34.1) | 2,153 (32.9) | 2,798 (31.8) |  |
| 1-4 times per month | 3,773 (31.9) | 1,675 (37.9) | 2,403 (41.6) |  | 5,905 (34.0) | 1,047 (43.8) | 899 (40.4) |  | 2,903 (33.0) | 2,134 (37.8) | 2,814 (37.7) |  |
| More than 5 times per month | 2,712 (22.1) | 959 (21.4) | 1,412 (23.0) |  | 3,863 (21.7) | 606 (24.1) | 614 (24.1) |  | 2,082 (22.5) | 1,259 (21.8) | 1,742 (22.2) |  |
| Hypertension |  |  |  |  |  |  |  |  |  |  |  |  |
| Yes | 4,802 (30.8) | 1,309 (23.4) | 1,554 (20.7) | <.001** | 6,230 (27.3) | 567 (19.0) | 868 (28.3) | <.001** | 3,287 (27.7) | 1,791 (24.5) | 2,587 (26.3) | 0.003** |
| No | 8,352 (69.2) | 3,412 (76.6) | 4,680 (79.3) |  | 12,853 (72.7) | 1,980 (81.0) | 1,611 (71.7) |  | 6,457 (72.3) | 4,353 (75.5) | 5,634 (73.7) |  |
| Diabetes |  |  |  |  |  |  |  |  |  |  |  |  |
| Yes | 1,888 (12.0) | 532 (9.3) | 581 (7.4) | <.001** | 2,486 (10.7) | 187 (6.1) | 328 (10.8) | <.001** | 1,309 (10.9) | 677 (9.0) | 1,015 (10.2) | 0.001** |
| No | 11,266 (88.0) | 4,189 (90.7) | 5,653 (92.6) |  | 16,597 (89.3) | 2,360 (93.9) | 2,151 (89.2) |  | 8,435 (89.1) | 5,467 (91.0) | 7,206 (89.8) |  |
| Musculoskeletal Disease |  |  |  |  |  |  |  |  |  |  |  |  |
| Yes | 2,447 (13.9) | 617 (9.5) | 568 (6.3) | <.001** | 3,126 (11.9) | 212 (5.6) | 294 (8.3) | <.001** | 1,672 (12.3) | 878 (10.0) | 1,082 (9.7) | <.001** |
| No | 10,707 (86.2) | 4,104 (90.5) | 5,666 (93.7) |  | 15,957 (88.1) | 2,335 (94.4) | 2,185 (91.7) |  | 8,072 (87.7) | 5,266 (90.0) | 7,139 (90.3) |  |
| Hypercholesterolemia |  |  |  |  |  |  |  |  |  |  |  |  |
| Yes | 3,184 (21.5) | 1,006 (18.2) | 1,138 (15.9) | <.001** | 4,307 (19.8) | 477 (16.3) | 544 (18.6) | 0.003** | 2,167 (19.6) | 1,333 (18.9) | 1,828 (19.1) | 0.624** |
| No | 9,970 (78.5) | 3,715 (81.8) | 5,096 (84.1) |  | 14,776 (80.2) | 2,070 (83.7) | 1,935 (81.4) |  | 7,577 (80.4) | 4,811 (81.1) | 6,393 (80.9) |  |
| Hypertriglyceridemia |  |  |  |  |  |  |  |  |  |  |  |  |
| Yes | 2,001 (15.7) | 642 (14.4) | 809 (13.5) | 0.002** | 2,800 (15.3) | 345 (14.2) | 307 (12.3) | 0.004** | 1,519 (16.3) | 841 (14.2) | 1,092 (13.7) | <.001** |
| No | 11,153 (84.3) | 4,079 (85.6) | 5,425 (86.5) |  | 16,283 (84.7) | 2,202 (85.8) | 2,172 (87.7) |  | 8,225 (83.7) | 5,303 (85.8) | 7,129 (86.3) |  |
| Data were expressed as the estimated mean ± standard error for continuous variables and frequency (weighted %) for categorical variables.  * P value using t-test.  ** P value using chi-square test.  † The means difference is not significant at the 0.05 level by Scheffe post-hoc test. | | | | | | | | | | | | |
