# Supplementary material for: Association between levels of physical activity and low handgrip strength: Korea National Health and Nutrition Examination Survey 2014-2019
Source: Epidemiol Health. 2022 Feb 21;44:e2022027. doi: 10.4178/epih.e2022027 (PMC9117110; doi:10.4178/epih.e2022027)
Supplement: Supplementary Material 2. — Multivariable logistic regression analysis on low handgrip strength associated with levels of physical activity [file epih-44-e2022027-suppl2.docx]

| **Supplementary Material 2. Multivariable logistic regression analysis on low handgrip strength associated with levels of physical activity** | | | | | | | | | |
| --- | --- | --- | --- | --- | --- | --- | --- | --- | --- |
|  | **Aerobic exercise*** | | | **Muscle strengthening exercise** | | | **Walking exercise*** | | |
|  | **Highly active** | **Active** | **Inactive** | **Highly active** | **Active** | **Inactive** | **Highly active** | **Active** | **Inactive** |
|  | **Odds Ratio (95% confidence interval)** | | | | | | | | |
| **Total** |  |  |  |  |  |  |  |  |  |
| Crude | 1 | 1.42  (1.27-1.59) | 1.60  (1.46-1.76) | 1 | 1.09  (0.92-1.30) | 1.66  (1.46-1.90) | 1 | 1.19  (1.08-1.30) | 1.28  (1.18-1.40) |
| Model 1 | 1 | 1.33  (1.19-1.49) | 1.40  (1.27-1.54) | 1 | 1.15  (0.96-1.37) | 1.71  (1.49-1.96) | 1 | 1.19  (1.08-1.31) | 1.19  (1.08-1.30) |
| Model 2 | 1 | 1.33  (1.19-1.49) | 1.38  (1.26-1.52) | 1 | 1.17  (0.98-1.39) | 1.68  (1.46-1.93) | 1 | 1.19  (1.08-1.31) | 1.16  (1.06-1.27) |
| Model 3 | 1 | 1.33  (1.18-1.49) | 1.37  (1.25-1.51) | 1 | 1.17  (0.98-1.39) | 1.68  (1.46-1.93) | 1 | 1.19  (1.08-1.30) | 1.16  (1.06-1.27) |
| Men^1^ |  |  |  |  |  |  |  |  |  |
| Crude | 1 | 1.48  (1.26-1.74) | 1.49  (1.30-1.70) | 1 | 1.17  (0.94-1.46) | 1.72  (1.45-2.04) | 1 | 1.14  (0.99-1.31) | 1.10  (0.96-1.25) |
| Model 1 | 1 | 1.37  (1.16-1.62) | 1.28  (1.12-1.47) | 1 | 1.21  (0.97-1.51) | 1.72  (1.45-2.05) | 1 | 1.13  (0.98-1.31) | 1.00  (0.87-1.14) |
| Model 2 | 1 | 1.37  (1.16-1.62) | 1.28  (1.11-1.47) | 1 | 1.25  (0.99-1.56) | 1.74  (1.46-2.08) | 1 | 1.14  (0.99-1.32) | 0.99  (0.86-1.13) |
| Model 3 | 1 | 1.36  (1.15-1.61) | 1.27  (1.11-1.46) | 1 | 1.25  (0.99-1.57) | 1.74  (1.46-2.07) | 1 | 1.14  (0.99-1.32) | 0.99  (0.86-1.13) |
| Women^1^ |  |  |  |  |  |  |  |  |  |
| Crude | 1 | 1.40  (1.21-1.63) | 1.76  (1.55-1.99) | 1 | 0.97  (0.73-1.30) | 1.62  (1.29-2.03) | 1 | 1.24  (1.10-1.41) | 1.50  (1.34-1.67) |
| Model 1 | 1 | 1.33  (1.14-1.55) | 1.54  (1.35-1.75) | 1 | 1.03  (0.77-1.38) | 1.65  (1.31-2.07) | 1 | 1.24  (1.09-1.41) | 1.39  (1.24-1.55) |
| Model 2 | 1 | 1.33  (1.14-1.55) | 1.50  (1.32-1.71) | 1 | 1.04  (0.78-1.39) | 1.58  (1.25-1.99) | 1 | 1.24  (1.09-1.41) | 1.36  (1.21-1.52) |
| Model 3 | 1 | 1.32  (1.13-1.55) | 1.50  (1.31-1.71) | 1 | 1.04  (0.78-1.39) | 1.58  (1.26-1.99) | 1 | 1.24  (1.09-1.41) | 1.36  (1.21-1.52) |
| **Age groups** |  |  |  |  |  |  |  |  |  |
| 19-39 |  |  |  |  |  |  |  |  |  |
| Crude | 1 | 1.52  (1.27-1.83) | 1.33  (1.13-1.58) | 1 | 1.15  (0.83-1.60) | 1.74  (1.33-2.27) | 1 | 1.15  (0.97-1.36) | 1.11  (0.93-1.32) |
| Model 1 | 1 | 1.44  (1.19-1.73) | 1.18  (0.99-1.42) | 1 | 1.17  (0.84-1.63) | 1.86  (1.41-2.45) | 1 | 1.12  (0.94-1.33) | 1.00  (0.84-1.20) |
| Model 2 | 1 | 1.44  (1.19-1.74) | 1.17  (0.97-1.40) | 1 | 1.21  (0.86-1.68) | 1.94  (1.47-2.57) | 1 | 1.12  (0.94-1.33) | 0.97  (0.81-1.17) |
| Model 3 | 1 | 1.43  (1.18-1.73) | 1.17  (0.97-1.40) | 1 | 1.20  (0.86-1.68) | 1.93  (1.46-2.57) | 1 | 1.13  (0.95-1.34) | 0.98  (0.82-1.18) |
| 40-59 |  |  |  |  |  |  |  |  |  |
| Crude | 1 | 1.45  (1.21-1.73) | 1.66  (1.44-1.92) | 1 | 1.03  (0.78-1.35) | 1.33  (1.08-1.65) | 1 | 1.25  (1.08-1.45) | 1.26  (1.10-1.46) |
| Model 1 | 1 | 1.41  (1.18-1.69) | 1.58  (1.36-1.83) | 1 | 1.05  (0.80-1.39) | 1.38  (1.12-1.71) | 1 | 1.27  (10.9-1.48) | 1.24  (1.07-1.44) |
| Model 2 | 1 | 1.40  (1.17-1.68) | 1.55  (1.33-1.80) | 1 | 1.08  (0.82-1.43) | 1.34  (1.08-1.67) | 1 | 1.28  (1.10-1.49) | 1.22  (1.05-1.41) |
| Model 3 | 1 | 1.40  (1.17-1.67) | 1.54  (1.32-1.79) | 1 | 1.09  (0.83-1.44) | 1.35  (1.09-1.67) | 1 | 1.27  (1.09-1.48) | 1.22  (1.05-1.41) |
| 60+ |  |  |  |  |  |  |  |  |  |
| Crude | 1 | 1.17  (0.95-1.43) | 1.72  (1.46-2.03) | 1 | 1.10  (0.79-1.52) | 2.18  (1.78-2.68) | 1 | 1.18  (1.01-1.38) | 1.55  (1.35-1.77) |
| Model 1 | 1 | 1.05  (0.85-1.29) | 1.43  (1.21-1.70) | 1 | 1.17  (0.83-1.64) | 2.14  (1.73-2.64) | 1 | 1.14  (0.97-1.34) | 1.33  (1.15-1.53) |
| Model 2 | 1 | 1.03  (0.84-1.27) | 1.35  (1.14-1.60) | 1 | 1.19  (0.85-1.65) | 1.92  (1.54-2.39) | 1 | 1.15  (0.98-1.35) | 1.26  (1.09-1.45) |
| Model 3 | 1 | 1.03  (0.83-1.26) | 1.33  (1.12-1.59) | 1 | 1.20  (0.86-1.67) | 1.91  (1.53-2.37) | 1 | 1.15  (0.98-1.35) | 1.25  (1.09-1.44) |

The data were presented as odds ratio (95% confidence interval).

Model 1: adjusted for age, BMI.

Model 2: further adjusted for education level, household income, smoking status, frequency of alcohol drinking.

Model 3: further adjusted for comorbidities (hypertension, diabetes, musculoskeletal disease, hypercholesterolemia, and hypertriglyceridemia).

*We adjusted muscle strengthening exercise which could be influenced on an association between physical activity and handgrip strength.
