# Supplementary material for: Association between levels of physical activity and low handgrip strength: Korea National Health and Nutrition Examination Survey 2014-2019
Source: Epidemiol Health. 2022 Feb 21;44:e2022027. doi: 10.4178/epih.e2022027 (PMC9117110; doi:10.4178/epih.e2022027)
Supplement: Supplementary Material 3. — Multivariable logistic regression analysis on low handgrip strength associated with levels of physical activity in men [file epih-44-e2022027-suppl3.docx]

| **Supplementary Material 3. Multivariable logistic regression analysis on low handgrip strength associated with levels of physical activity in men** | | | | | | | | | |
| --- | --- | --- | --- | --- | --- | --- | --- | --- | --- |
|  | **Aerobic exercise*** | | | **Muscle strengthening exercise** | | | **Walking exercise*** | | |
|  | **Highly active** | **Active** | **Inactive** | **Highly active** | **Active** | **Inactive** | **Highly active** | **Active** | **Inactive** |
|  | **Odds Ratio (95% confidence interval)** | | | | | | | | |
| **Age groups** |  |  |  |  |  |  |  |  |  |
| 19-39 |  |  |  |  |  |  |  |  |  |
| Crude | 1 | 1.49  (1.16-1.93) | 1.26  (1.00-1.60) | 1 | 1.08  (0.73-1.60) | 1.78  (1.29-2.46) | 1 | 1.08  (0.84-1.38) | 0.96  (0.74-1.23) |
| Model 1 | 1 | 1.35  (1.04-1.75) | 1.07  (0.83-1.38) | 1 | 1.08  (0.73-1.61) | 1.83  (1.31-2.5) | 1 | 1.04  (0.81-1.34) | 0.86  (0.66-1.12) |
| Model 2 | 1 | 1.33  (1.01-1.74) | 1.07  (0.82-1.39) | 1 | 1.13  (0.76-1.68) | 2.02  (1.44-2.85) | 1 | 1.05  (0.81-1.36) | 0.83  (0.63-1.09) |
| Model 3 | 1 | 1.32  (1.00-1.73) | 1.07  (0.82-1.38) | 1 | 1.13  (0.75-1.68) | 2.01  (1.43-2.84) | 1 | 1.05  (0.81-1.37) | 0.83  (0.63-1.10) |
| 40-59 |  |  |  |  |  |  |  |  |  |
| Crude | 1 | 1.59  (1.22-2.06) | 1.53  (1.24-1.88) | 1 | 1.18  (0.82-1.70) | 1.31  (0.98-1.75) | 1 | 1.12  (0.89-1.42) | 1.04  (0.84-1.29) |
| Model 1 | 1 | 1.57  (1.21-2.05) | 1.45  (1.17-1.80) | 1 | 1.22  (0.84-1.76) | 1.33  (0.99-1.78) | 1 | 1.14  (0.90-1.44) | 1.01  (0.81-1.26) |
| Model 2 | 1 | 1.55  (1.19-2.02) | 1.44  (1.16-1.79) | 1 | 1.23  (0.84-1.79) | 1.31  (0.98-1.76) | 1 | 1.16  (0.92-1.46) | 1.01  (0.81-1.26) |
| Model 3 | 1 | 1.55  (1.18-2.02) | 1.44  (1.16-1.79) | 1 | 1.25  (0.86-1.82) | 1.33  (0.99-1.78) | 1 | 1.14  (0.90-1.44) | 1.00  (0.80-1.25) |
| 60+ |  |  |  |  |  |  |  |  |  |
| Crude | 1 | 1.31  (0.99-1.73) | 1.76  (1.41-2.20) | 1 | 1.22  (0.81-1.84) | 2.41  (1.88-3.09) | 1 | 1.31  (1.05-1.64) | 1.47  (1.21-1.79) |
| Model 1 | 1 | 1.15  (0.86-1.54) | 1.43  (1.14-1.80) | 1 | 1.27  (0.83-1.95) | 2.30  (1.79-2.96) | 1 | 1.27  (1.01-1.61) | 1.26  (1.02-1.54) |
| Model 2 | 1 | 1.12  (0.83-1.50) | 1.32  (1.05-1.66) | 1 | 1.28  (0.84-1.95) | 2.03  (1.56-2.64) | 1 | 1.30  (1.03-1.65) | 1.15  (0.93-1.41) |
| Model 3 | 1 | 1.11  (0.83-1.49) | 1.31  (1.04-1.65) | 1 | 1.28  (0.84-1.96) | 2.00  (1.53-2.60) | 1 | 1.30  (1.03-1.65) | 1.14  (0.93-1.40) |

The data were presented as odds ratio (95% confidence interval).

Model 1: adjusted for age, BMI.

Model 2: further adjusted for education level, household income, smoking status, frequency of alcohol drinking.

Model 3: further adjusted for comorbidities (hypertension, diabetes, musculoskeletal disease, hypercholesterolemia, and hypertriglyceridemia).

*We adjusted muscle strengthening exercise which could be influenced on an association between physical activity and handgrip strength.
