# Supplementary material for: Association between levels of physical activity and low handgrip strength: Korea National Health and Nutrition Examination Survey 2014-2019
Source: Epidemiol Health. 2022 Feb 21;44:e2022027. doi: 10.4178/epih.e2022027 (PMC9117110; doi:10.4178/epih.e2022027)
Supplement: Supplementary Material 4. — Multivariable logistic regression analysis on low handgrip strength associated with levels of physical activity in women [file epih-44-e2022027-suppl4.docx]

| **Supplementary Material 4. Multivariable logistic regression analysis on low handgrip strength associated with levels of physical activity in women** | | | | | | | | | |
| --- | --- | --- | --- | --- | --- | --- | --- | --- | --- |
|  | **Aerobic exercise*** | | | **Muscle strengthening exercise** | | | **Walking exercise*** | | |
|  | **Highly active** | **Active** | **Inactive** | **Highly active** | **Active** | **Inactive** | **Highly active** | **Active** | **Inactive** |
|  | **Odds Ratio (95% confidence interval)** | | | | | | | | |
| **Age groups** |  |  |  |  |  |  |  |  |  |
| 19-39 |  |  |  |  |  |  |  |  |  |
| Crude | 1 | 1.67  (1.29-2.16) | 1.52  (1.19-1.94) | 1 | 1.53  (0.82-2.85) | 2.16  (1.25-3.70) | 1 | 1.25  (0.98-1.59) | 1.31  (1.04-1.65) |
| Model 1 | 1 | 1.61  (1.24-2.09) | 1.35  (1.05-1.75) | 1 | 1.48  (0.79-2.77) | 2.13  (1.24-3.66) | 1 | 1.22  (0.96-1.55) | 1.20  (0.95-1.52) |
| Model 2 | 1 | 1.63  (1.25-2.11) | 1.34  (1.04-1.73) | 1 | 1.47  (0.80-2.71) | 2.08  (1.22-3.55) | 1 | 1.22  (0.96-1.55) | 1.18  (0.93-1.50) |
| Model 3 | 1 | 1.62  (1.25-2.11) | 1.36  (1.05-1.75) | 1 | 1.47  (0.80-2.73) | 2.09  (1.22-3.58) | 1 | 1.24  (0.97-1.58) | 1.20  (0.95-1.52) |
| 40-59 |  |  |  |  |  |  |  |  |  |
| Crude | 1 | 1.35  (1.07-1.70) | 1.80  (1.49-2.18) | 1 | 0.82  (0.54-1.24) | 1.34  (0.97-1.85) | 1 | 1.37  (1.14-1.66) | 1.53  (1.28-1.82) |
| Model 1 | 1 | 1.31  (1.03-1.66) | 1.70  (1.40-2.06) | 1 | 0.84  (0.56-1.28) | 1.39  (1.01-1.92) | 1 | 1.40  (1.15-1.69) | 1.50  (1.26-1.79) |
| Model 2 | 1 | 1.29  (1.01-1.63) | 1.66  (1.36-2.02) | 1 | 0.88  (0.58-1.33) | 1.33  (0.96-1.85) | 1 | 1.40  (1.15-1.69) | 1.47  (1.23-1.76) |
| Model 3 | 1 | 1.29  (1.01-1.64) | 1.65  (1.35-2.01) | 1 | 0.88  (0.58-1.34) | 1.34  (0.97-1.87) | 1 | 1.40  (1.15-1.69) | 1.47  (1.23-1.76) |
| 60+ |  |  |  |  |  |  |  |  |  |
| Crude | 1 | 1.03  (0.77-1.39) | 1.64  (1.29-2.08) | 1 | 0.86  (0.50-1.50) | 1.81  (1.24-2.63) | 1 | 1.07  (0.86-1.34) | 1.59  (1.32-1.91) |
| Model 1 | 1 | 0.95  (0.71-1.28) | 1.41  (1.10-1.81) | 1 | 0.95  (0.54-1.65) | 1.76  (1.21-2.56) | 1 | 1.04  (0.83-1.31) | 1.37  (1.14-1.66) |
| Model 2 | 1 | 0.96  (0.72-1.30) | 1.37  (1.07-1.76) | 1 | 0.97  (0.55-1.69) | 1.63  (1.12-2.38) | 1 | 1.06  (0.84-1.33) | 1.35  (1.11-1.64) |
| Model 3 | 1 | 0.97  (0.72-1.30) | 1.35  (1.05-1.74) | 1 | 0.99  (0.56-1.73) | 1.62  (1.11-2.36) | 1 | 1.05  (0.84-1.32) | 1.35  (1.11-1.63) |

The data were presented as odds ratio (95% confidence interval).

Model 1: adjusted for age, BMI.

Model 2: further adjusted for education level, household income, smoking status, frequency of alcohol drinking.

Model 3: further adjusted for comorbidities (hypertension, diabetes, musculoskeletal disease, hypercholesterolemia, and hypertriglyceridemia).

*We adjusted muscle strengthening exercise which could be influenced on an association between physical activity and handgrip strength.
