# Supplementary figures and images for: Association between levels of physical activity and low handgrip strength: Korea National Health and Nutrition Examination Survey 2014-2019
Source: Epidemiol Health. 2022 Feb 21;44:e2022027. doi: 10.4178/epih.e2022027 (PMC9117110; doi:10.4178/epih.e2022027)

**Supplementary Ma
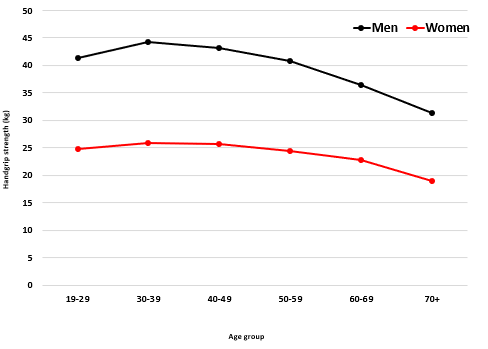
terial 5. Mean handgrip strength by age groups in men and women.**

Supplement: Supplementary Material 5. — Mean handgrip strength by age groups in men and women. [file epih-44-e2022027-suppl5.docx]
