# Supplementary material for: Association between levels of physical activity and low handgrip strength: Korea National Health and Nutrition Examination Survey 2014-2019
Source: Epidemiol Health. 2022 Feb 21;44:e2022027. doi: 10.4178/epih.e2022027 (PMC9117110; doi:10.4178/epih.e2022027)
Supplement: Supplementary file 6 [file epih-44-e2022027-suppl.docx]

**Association between levels of physical activity and low handgrip strength: Korea National Health and Nutrition Examination Survey 2014-2019**

안형순, 최화영, 기모란

국립암센터 국제암대학원대학교 암관리학과

**Abstract**

Objectives: This study aimed to investigate the association between levels of physical activity (PA) and low handgrip strength in Korean adults.

Methods: Our cross-sectional study design included 24,109 Korean adults older than 19 years of age who participated in the Korea National Health and Nutrition Examination Survey 2014–2019. Low handgrip strength is described as hand strength less than the cut-off value of the 20th percentile of handgrip strength from a healthy population in each gender and age group. PA was categorized into three levels (inactive, active, and highly active) according to the World Health Organization’s global recommendations on PA for health. Multivariable logistic regression analysis was used to examine the association between levels of PA and low handgrip strength.

Results: Odds ratios (ORs) for low handgrip strength were significantly higher in middle-aged women who were active (adjusted odds ratio [aOR], 1.40; 95% confidence interval [CI], 1.15 to 1.69) and inactive (aOR, 1.47; 95% CI, 1.23 to 1.76) than in those highly active in walking exercise. Most of older people had significantly higher ORs for low handgrip strength in active compared to highly active in the context of aerobic, muscle strengthening, and walking exercise.

Conclusions: Walking exercise was associated with a lower risk of sarcopenia in middle-aged women and older individuals. However, further studies are necessary to confirm the causal relationship between levels of PA and low handgrip strength.

**Keywords:** Sarcopenia, Hand strength, Physical activity, Korean adults

**Introduction**

근감소증은 낙상, 골절, 신체적 장애 및 사망과 관련된 진행성 질환이며 일반적으로 골격근량과 근력의 저하가 동반된 골격근 장애이다 [1]. 그 동안 노화로 인한 자연스러운 현상으로 여겨졌던 근감소증은 2016년 ICD-10-CM (International Classification of Diseases, Tenth Revision, Clinical Modification)에 질병코드가 부여되면서 하나의 질병으로 인식되기 시작하였다 [2]. 근감소증의 진단은 과학적 근거를 바탕으로 정기적으로 업데이트 되었다. 2010년 European Working Group on Sarcopenia in Older People (EWGSOP1)은 근감소증의 진단을 위해 근육량 저하를 우선적으로 활용하였다면, 2018년 EWGSOP2는 근력이 근육량 보다 근감소증의 이상징후(adverse outcomes)를 예측하는데 더 우수하다는 과학적 근거를 바탕으로 근력 저하가 근감소증의 진단에 우선됨을 제안하였다 [1, 3]. EWGSOP2는 먼저 근력 저하로 근감소증의 가능성을 식별하고 근육량 저하(low muscle quantity) 또는 근육의 질 저하(low muscle quality)를 통해 근감소증을 확정, 추가적으로 신체 기능 저하(low physical performance)를 측정하여 근감소증의 중증도를 확인하는 방식을 통해 근감소증의 진단을 보다 체계적으로 확립하였다 [1]. 또한 EWGSOP2는 근감소증의 새로운 진단 기준과 함께 근감소증이 보다 젊은 층에서부터 시작될 수 있음을 강조하고 있다 [1]. 이는 노년층에서 발생하는 근감소증을 예방 및 지연시키기 위하여 젊은 시절부터 근육량과 근력을 관리할 필요가 있음이 강조되고 있는 것이다. 젊었을 때부터 근감소증의 이차적 요인인 질병과 영양부족, 신체활동 부족을 관리한다면 노년기에 발생하는 근감소증을 예방하고 지연시킬 수 있다는 것을 의미한다. EWGSOP2의 새로운 근감소증 진단 알고리즘에 따라 근력 저하를 통한 근감소증의 진단에 악력을 활용할 수 있으며, 임상 실습 또는 진료실에서 기준값(men: 27kg, women: 16kg) 미만의 낮은 악력을 나타낸 대상자에게 근감소증을 개선하기 위한 중재를 시작하기에 충분하다고 정의한다 [1].

근감소증의 중재 방법 중 신체활동의 중요성은 다수의 선행 연구를 통해 확인할 수 있다 [4-7]. 저항성 운동(resistance exercise)은 대표적인 근감소증 예방 및 치료와 연관된 신체활동으로 특히 상지 근력을 단련하는 상체 위주의 운동이 악력 증진에 긍정적인 효과가 있는 것으로 나타났다 [8]. 또한 저항성 운동과 더불어 균형성, 유연성 및 지구력 운동을 복합적으로 시행할 경우에 악력 향상에 더욱 도움이 될 수 있다는 분석도 있다 [9]. 그러나 이러한 연구의 대부분은 노년층의 신체활동과 악력의 연관성에만 초점을 맞추고 있어 근감소증의 선제적 관리 차원의 젊은 층을 포함한 근감소증 중재프로그램에 대한 근거는 제공하지 못하는 실정이다. Lee et al.은 우리나라 만 10세 이상을 대상으로 성별에 상관없이 중년의 시기부터 악력이 감소하기 시작한다는 연구 결과를 발표했으며, 우리나라 성인을 대상으로 한 Kim et al.의 연구에서도 남녀 모두 40대부터 악력의 평균값이 감소함을 알 수 있었다 [10, 11]. 두 연구 모두 단면연구(cross-sectional study)로 40대의 악력 감소가 노년층에서의 근감소증에 미치는 영향을 알기엔 부족하지만 악력이 감소하는 중년부터 근감소증을 예방하는 차원에서의 신체활동이 필요함을 시사한다. Dodds et al.의 연구도 중년층부터 축적된 근육이 노년층의 근육 감소를 대비할 수 있다는 연구 결과를 나타내어 중년층의 활발한 신체활동을 권장하고 있다 [12].

지금까지 노년층 한국인을 대상으로 한 신체활동과 악력과의 연관성을 분석한 연구는 있었으나, 한국인 만 19세 이상 성인 전체를 대상으로 신체활동과 낮은 악력과의 관계를 분석한 연구는 없었다 [13]. 이에 본 연구는 국민건강영양조사 2014-2019년도 자료를 활용하여 한국인 만 19세 이상 건강한 성인을 대상으로 낮은 악력의 기준값을 성별 및 연령그룹별로 도출하고 유산소성 운동, 근력 운동, 걷기 운동 수준과 낮은 악력의 연관성을 분석하고자 한다.

**Methods**

**Data collection and Study participants**

본 연구는 2014-2019년 국민건강영양조사를 활용하여 조사에 참여한 45,022명 중 만 19세 이상으로 주로 쓰는 손의 악력이 3회 측정된 33,687명을 분석 대상으로 선정하였다. 이 중 키, 몸무게, BMI, 학력 수준, 가구소득 수준, 흡연, 음주빈도, 동반 질환(고혈압, 당뇨병, 근골격계 질환, 고콜레스테롤혈증, 고중성지질혈증)과 신체활동(유산소성 운동, 근력 운동, 걷기 운동)에 응답한 24,109명을 최종 분석 대상으로 선정하였다. 모든 조사 참여 대상자에게 서면 동의서를 제공했으며, 질병관리청의 연구윤리위원회의 승인을 받았다 (IRB number: 2013-12EXP-03-5C, 2018-01-03-P-A, 2018-01-03-C-A).

**Handgrip strength and cutoff values**

국민건강영양조사는 2014년부터 10세 이상을 대상으로 디지털 악력계(Digital grip strength dynamometer, T.K.K 5401, Japan)를 이용하여 악력 검사를 시행하고 있다. 본 연구에서는 주로 쓰는 손의 3회 측정값 중 최대 악력값을 분석에 활용하였고, 양손잡이의 경우 6회 측정값 중 최대 악력값을 분석에 활용하였다. 낮은 악력의 기준값은 건강한 인구 집단을 성별과 연령 그룹(19-29, 30-39, 40-49, 50-59, 60-69, 70+)으로 나누어 악력값의 하위 20백분위 값으로 정의하였다 [14]. 건강한 인구집단은 주로 쓰는 손의 악력을 3회 측정한 사람 중 국민건강영양조사의 활동제한 및 삶의 질 관련 설문의 ‘현재 건강상의 문제나 신체 혹은 정신적인 장애로 일상생활 및 사회활동에 제한을 받고 계십니까?’란 질문에 ‘아니오’라고 응답한 사람 중 건강관련 삶의 질 측정 도구인 EQ-5D의 다섯 가지 설문(운동능력, 자기관리, 일상활동, 통증/불편, 불안/우울)에 ‘지장이 없음’으로 응답한 자들로 선정하였다(n=21,462) [15]. 최종적으로 성별과 연령을 고려한 낮은 악력의 기준값은 남성의 경우, 19-29세는 35.7 kg, 30-39세는 38.2 kg, 40-49세는 37.4 kg, 50-59세는 36.1 kg, 60-69세는 33 kg, 70세 이상의 연령에서는 27.3 kg이었다. 여성의 경우, 19-29세는 20.6 kg, 30-39세는 22 kg, 40-49세는 22 kg, 50-59세는 21.3 kg, 60-69세는 19.8 kg, 70세 이상의 연령에서는 16.1 kg이 낮은 악력의 기준값이 되었다.

**Physical activity**

신체활동은 국민건강영양조사에서 만 19세 이상의 성인에게 제공되는 국제 신체활동 설문(Global Physical Activity Questionnaire, GPAQ)의 자기 보고식 설문 응답을 활용하여 측정하였으며, 본 연구는 신체활동 유형을 크게 유산소성 운동, 걷기 운동, 근력 운동으로 나누어 분석하였다. 유산소성 운동은 일 관련, 이동 관련, 여가 관련 신체활동 시간(분)을 사용하여 운동 강도를 고려한 신체활동 대사량(Metabolic Equivalent Task, MET)을 계산하였다. 일과 여가 관련 신체활동은 세계보건기구의 신체활동지침에 따라 고강도 신체활동엔 8 MET, 중강도 신체활동엔 4 MET을 곱하여 주당 신체활동 대사량(MET-mins/week)을 계산하였다 [16]. 이동 관련 신체활동엔 중강도와 동일한 4 MET을 곱하여 주당 신체활동 대사량(MET-mins/week)을 계산하였다. 걷기 운동도 WHO의 가이드라인을 활용하여 신체활동 시간(분)에 4 MET을 곱하여 주당 신체활동의 대사량(MET-mins/week)을 계산하였다 [16]. 세계보건기구의 신체활동지침을 참고하여 유산소성 운동과 걷기 운동은 주당 <600 MET minutes, 600≤MET minutes<1,200, 1,200≤MET minutes을 실천한 그룹을 각각 비활동적인 그룹(Inactive), 활동적인 그룹(Active), 매우 활동적인 그룹(Highly active)으로 분류하였다. 근력 운동은 주당 <2 days, 2-3 days, ≤4 days 실천한 그룹을 각각 비활동적인 그룹(Inactive), 활동적인 그룹(Active), 매우 활동적인 그룹(Highly active)으로 분류하였다.

**Covariates**

선행 연구들을 통하여 낮은 악력에 영향을 미치는 인구사회학적 요소로 체질량지수(BMI, 저체중: BMI<18.5, 정상: 18.5≤BMI<23.0, 과체중: 23.0≤BMI<25.0, 비만: 25.0≤BMI), 학력 수준(초등학교 이하, 중학교, 고등학교, 대학 이상), 가구소득 수준(하, 중하, 중상, 상)을 사용하였다. 건강행태 관련 요소로 흡연(피운 적 없음, 과거 흡연, 현재 흡연), 음주 빈도(마신 적 없음, 한 달에 1회 미만, 한 달에 1~4회, 한 달에 5회 이상 음주)를 사용하였다 [11, 17-23]. 동반질환은 고혈압, 당뇨, 근골격계 질환, 고콜레스테롤혈증, 고중성지질혈증을 가진 환자로 정의하였다. 고혈압의 기준은 수축기 혈압이 140 mmHg 이상, 이완기 혈압이 90 mmHg 이상이며 항고혈압제 복용에 ‘예’ 라고 응답한 자로 하였다. 당뇨의 기준은 공복혈당이 126 mg/dL 이상이며 인슐린 주사를 맞거나 혈당강하약 복용, 의사에게 당뇨로 진단된 자로 하였다. 근골격계 질환의 기준은 관절염, 골관절염, 류머티스성 관절염, 골다공증 중 한가지라도 가지고 있는 자로 하였다. 고콜레스테롤혈증의 기준은 전체 콜레스테롤 수치가 240 mg/dL 이거나 콜레스테롤 저하제를 복용하고 있는 자로 하였으며, 고중성지질혈증은 12 시간의 공복 중 트리글리세리드 수치가 200 mg/dL 이상인 자로 하였다.

**Statistical analysis**

모든 통계적 분석은 다단계 층화 집락 추출(stratified multi-stage clustered sampling weight)에 의하여 생성된 가중치를 사용하여 분석되었다. 낮은 악력값에 관한 데이터는 가중치가 적용된 비율과 표준오차로 표시되었다. 범주형 변수에 관한 p값을 계산하기 위해 카이제곱 검정과 일원분산분석(one-way ANOVA)을 사용하였으며, 연속형 변수에 관한 p값의 계산을 위해 T검정을 사용하였다. 신체활동 수준과 낮은 악력의 연관성을 분석하기 위하여 다변수 로지스틱 회귀분석(multivariable logistic regression analysis)을 활용하여 highly active 신체활동 수준을 기준(reference)으로 하여 신체활동 수준이 active 및 inactive인 그룹의 낮은 악력의 유병오즈비(prevalence odds ratio)를 도출하였다. Model 1은 나이, 성별, 체질량지수를 보정하였다. Model 2는 Model 1에 추가적으로 학력 수준, 가구소득 수준, 흡연, 음주 빈도를 보정하였으며, Model 3은 Model 2에 추가적으로 동반질환(고혈압, 당뇨, 근골격계 질환, 고콜레스테롤혈증, 고중성지질혈증)을 보정하였다. 유산소성 및 걷기 운동에서 낮은 악력과의 연관성을 분석할 때, 근력 운동을 공변인으로 보정하여 보다 정확하게 신체활동 수준과 낮은 악력의 연관성을 분석하고자 하였다. 최종 분석 결과에는 무보정한 crude model 결과와 모든 공변인을 보정한 Model 3의 결과를 adjusted model로 명칭하여 기입하였다. 모든 분석에 국민건강영양조사 가이드라인에서 권장하는 복합 표본 설계를 반영하였다. SAS version 9.4 (SAS Institute Inc., Cary, NC, USA)를 사용하여 분석하였으며, 통계적 유의 수준은 p<0.05였다 (Supplementary Material 1-4).

**Results**

24,109명의 연구 대상자에 관한 특성은 Table 1에 제시되었다. 전체 연구 대상자 중 여성(51.2%)이 남성(48.8%)보다 많았으며 체질량지수가 과체중 이상인 그룹은 전체의 56.3%로 정상인 그룹(39.4%)보다 많았다. 가장 많은 동반질환은 고혈압(26.4%)이며 고콜레스테롤혈증(19.2%), 고중성지질혈증(14.8%), 근골격계 질환(10.8%), 당뇨병(10.1%) 순이었다. 모든 신체활동에서 inactive 그룹이 가장 많았으며, 근력 운동은 77.5%가 inactive 그룹(일주일에 2회 미만)에 속하였다. 유산소성 운동과 걷기 운동에서는 highly active 그룹의 비율이 active 그룹보다 많았다.

신체활동 수준과 관련된 요인은 Table 2에 요약되었다. 모든 신체활동에서 낮은 악력을 가진 그룹은 inactive 그룹에서 가장 많았다. 평균 악력값은 신체활동 수준이 올라갈 수록 증가하였으나, 사후검정 결과 걷기 운동의 inactive 그룹과 active 그룹의 평균 악력값은 유의한 차이가 없었다. 남성은 모든 신체활동에서 highly active 그룹이 가장 많았으며, 여성은 걷기 운동을 제외하고 inactive 그룹이 가장 많았다. 60대 이상 연령그룹은 근력운동에서 highly active 그룹이 가장 많은 특성을 나타냈다 (all p<0.05).

Table 3은 다변수 로지스틱 회귀분석을 통하여 신체활동 수준이 active 및 inactive한 그룹이 highly active한 그룹에 비하여 낮은 악력일 교차비(유병오즈비)를 나타낸 것이다. 전체 연구 대상자의 Adjusted model 분석결과, 유산소성 운동은 highly active 그룹에 비하여 active 그룹은 1.33배(aOR: 1.33, 95% CI: 1.18-1.49), inactive 그룹은 1.37배(aOR: 1.37, 95% CI: 1.25-1.51)로 낮은 악력의 유병오즈비가 유의하게 증가하였다. 근력 운동과 걷기 운동은 highly active 그룹에 비하여 inactive 그룹이 각각 1.68배(aOR: 1.68, 95% CI: 1.46-1.93), 1.16배(aOR: 1.16, 95% CI: 1.06-1.27)로 낮은 악력의 유병오즈비가 유의하게 증가하였다. 걷기 운동에서는 active 그룹이 highly active 그룹에 비하여 낮은 악력의 유병오즈비가 1.19배(aOR: 1.19, 95% CI: 1.08-1.30) 유의하게 증가하였다.

전체 연구 대상자를 성별로 나눈 Adjusted model 분석결과, 여성은 걷기 운동에서 낮은 악력의 유병오즈비가 highly active 그룹에 비하여 active 그룹은 1.24배(aOR: 1.24, 95% CI: 1.09-1.41), inactive 그룹은 1.36배(aOR: 1.36, 95% CI: 1.21-1.52)로 유의하게 증가했으나 남성은 걷기 운동에서 낮은 악력의 유병오즈비가 highly active 그룹과 active 및 inactive 그룹 간 유의한 차이가 없었다.

전체 연구대상자를 연령대(19-39세, 40-59세, 60세 이상)별로 나눈 Adjusted model 분석결과, 청년층(19-39세)의 낮은 악력의 유병오즈비는 유산소성 운동을 highly active하게 한 그룹에 비하여 active 그룹이 1.43배(aOR: 1.43, 95% CI: 1.18-1.73)로 유의하게 증가했으며, 근력 운동이 inactive한 그룹이 highly active 그룹보다 낮은 악력의 유병오즈비가 약 2배(aOR: 1.93, 95% CI: 1.46-2.57)가 높았다. 중년층(40-59세)의 낮은 악력의 유병오즈비는 유산소성 운동의 경우 highly active 그룹에 비하여 active 그룹이 1.40배(aOR: 1.40, 95% CI: 1.17-1.67), inactive 그룹이 1.54배(aOR: 1.54, 95% CI: 1.32-1.79)로 유의하게 증가하였다. 근력 운동과 걷기 운동은 inactive한 그룹에서 highly active 그룹에 비해 각각 1.35배(aOR: 1.35, 95% CI: 1.09-1.67), 1.22배(aOR: 1.22, 95% CI: 1.05-1.41)로 낮은 악력의 유병오즈비가 유의하게 증가하였으며, 걷기 운동의 active 그룹은 1.27배(aOR: 1.27, 95% CI: 1.09-1.48) 유의하게 증가하였다. 노년층(60세 이상)에서는 유산소성 운동, 근력 운동, 걷기 운동이 inactive한 그룹이 highly active한 그룹에 비해 낮은 악력의 유병오즈비가 각각 1.33배(aOR: 1.33, 95% CI: 1.12-1.59), 1.91배(aOR: 1.91, 95% CI: 1.53-2.37), 1.25배(aOR: 1.25, 95% CI: 1.09-1.44)로 유의하게 증가하였다. 청년층과 중년층, 노년층까지 근력 운동의 active 그룹은 highly active 그룹에 비해 낮은 악력의 유병오즈비가 증가하였으나 유의한 차이는 없었다.

Figure 1은 하위 집단 분석으로 전체 연구대상자를 신체활동별로 성별과 연령그룹별로 나누어 낮은 악력의 유병오즈비를 나타낸 것이다. 청년층 남성과 달리 청년층 여성은 유산소성 운동이 highly active한 그룹에 비해 inactive 그룹에서 낮은 악력의 유병오즈비가 1.36배(aOR: 1.36, 95% CI: 1.05-1.75), 중년층 여성의 경우 걷기 운동을 active 및 inactive한 그룹에서 highly active한 그룹에 비해 낮은 악력의 유병오즈비가 각각 1.40배(aOR: 1.40, 95% CI: 1.15-1.69), 1.47배(aOR: 1.47, 95% CI: 1.23-1.76) 유의하게 증가하였다. 노년층 여성의 경우, 모든 신체활동에서 inactive한 그룹이 highly active한 그룹에 비해 낮은 악력의 유병오즈비가 유의하게 증가하였다. 반면 노년층 남성의 경우에 유산소성 운동과 근력 운동에서는 inactive한 그룹이 highly active한 그룹보다 낮은 악력의 유병오즈비가 유의하게 증가하였으며, 걷기 운동에서는 active한 그룹이 highly active한 그룹보다 낮은 악력의 유병오즈비가 1.30배(aOR: 1.30, 95% CI:1.03-1.65)배 유의하게 증가하였다.

**Discussion**

본 연구는 한국인의 특성을 대표하는 국민건강영양조사 2014-2019년 자료를 활용하여 한국인 만 19세 이상 성인의 신체활동 수준과 낮은 악력의 연관성을 분석하였다. 신체활동은 유산소성 운동, 근력 운동, 걷기 운동의 3가지 유형으로 분류하여 연구 대상자들을 비활동적인 그룹(Inactive)과 활동적인 그룹(Active), 매우 활동적인 그룹(Highly active: reference)으로 나누었다. 만 19세 이상 연구 대상자 중 건강한 인구 집단을 성별, 연령 그룹별로 나누어 각각 하위 20번째 백분위 값을 기준으로 낮은 악력을 가진 그룹과 아닌 그룹을 구분하였다.

전체 연구대상자를 남녀로 구분하여 봤을 때, 남녀 간 주목할 만한 차이는 여성에게는 걷기 운동과 낮은 악력 간 유의한 연관성이 나타났으나 남성에서는 연관성이 없었다는 것이다. 특히 중년층 여성에게서 걷기 운동의 수준이 낮아질수록 낮은 악력의 유병오즈비가 유의하게 증가하였다. 이는 EWGSOP2의 근감소증 진단 가이드라인 중 낮은 악력이 근감소증을 진단하는데 우선시되는 지표임을 반영할 때, 걷기 운동이 한국인 여성에서 근감소증 발병을 예방하는 효과가 있을 가능성을 시사한다 [1]. 중년 여성의 걷기와 근력 및 근육량의 연관성에 관한 연구는 많지 않다. 걷기가 50세 이상 중고령 여성의 체성분(body composition) 변화에 미치는 영향에 관한 연구를 진행한 Gaba et al.은 Brisk Walking 중재 그룹(n=58)과 대조군(n=46)의 10주 후 체성분을 비교하였는데 두 그룹 간 유의미한 차이가 없었으며, Brisk Walking 중재 그룹 안에서 10주 전/후 체성분을 비교하였을 때, 하지제지방량(lean body mass of lower limbs, kg)은 유의하게 증가하였으나 상지제지방량(lean body mass of upper limbs, kg)은 유의한 차이가 없었다 [24]. 본 연구는 상지 근력 검사요소 중 하나인 악력과 걷기가 유의한 연관성이 있다는 분석 결과를 도출하여 Gaba et al.의 연구 결과와 차이를 나타낸다. 이러한 차이는 아마도 다음과 같은 이유 때문일 수 있다. Gaba et al.의 연구에서는 주로 사무실에서 좌식 생활을 하는 중고령 여성들을 대상으로 분석하였으며, 중재 기간도 평균 10주 정도만 진행되어 본 연구가 일반인을 대상으로 일상생활에서 걷기와 악력의 연관성을 본 것과는 차이가 있다. 또한 본 연구는 걷기 운동량 측정에 자기기입식 설문 결과를 활용하였고, Gaba et al.의 연구에서는 직접 스텝수를 측정하여 걷기 운동량을 도출한 차이가 있었다. 남녀 간 걷기 운동과 악력과의 연관성에 대한 차이는 아마도 남성과 여성의 체성분 차이 때문일 가능성이 있다. 남성이 여성보다 상지 근육량이 많기 때문에 남성에게 걷기 운동보다는 유산소성 운동이나 근력운동이 악력과 연관성이 있을 수 있다 [25].

연령대별로 보았을 때, 청년층의 경우에 남녀 모두 근력 운동의 비활동적인 수준이 매우 활동적인 수준보다 낮은 악력의 유병오즈비가 약 2배 정도 유의하게 증가하여 청년층에서 근력 운동과 낮은 악력간 높은 연관성이 있음을 알 수 있다. 그러나 청년층에서 걷기 운동과 낮은 악력은 유의한 연관성이 없는 것으로 나타났다. 본 연구 결과, 악력은 20대부터 30대까지 증가 추세를 나타내다가 40대부터 감소하기 시작하는 것을 알 수 있다(Supplementary material 5). 이는 높은 수준의 근력을 가진 20-30대 청년층에게 다른 신체활동 보다 근력 운동의 실천이 청년층의 근감소증 발생을 예방하는데 도움을 줄 가능성이 있음을 시사한다.

본 연구 결과, 중년층 남녀 모두에게 유산소성 운동이 활동적, 비활동적인 그룹은 매우 활동적인 그룹 보다 낮은 악력의 유병오즈비가 유의하게 증가하였다. 이는 Seung et al.이 국민건강영양조사 2014-2017년 자료를 활용하여 한국인 만 19세 이상 성인에서 유산소성 운동(활동: ≥600 MET-min/week, 비활동: <600 MET-min/week)을 비활동적으로 한 그룹이 활동적으로 한 그룹보다 낮은 악력의 유병 오즈비가 유의하게 높음을 분석한 연구와 유사한 결과이다 [26]. 이는 유산소성 운동이 중년층에서의 근감소증을 예방의 가능성이 있음을 나타낸다.

노년층 남녀 모두 걷기 운동이 낮은 악력의 유병오즈비와 연관이 있음을 알 수 있다. 단 노년층 여성의 경우, 매우 활동적인 그룹보다 비활동적인 그룹에서 낮은 악력의 유병오즈비가 유의하게 증가하였으나, 노년층 남성에서는 매우 활동적인 그룹보다 활동적인 그룹에서 낮은 악력의 유병오즈비가 유의하게 증가하였다. 이는 걷기 운동이 노년층 남녀의 근감소증 발생 위험을 감소시키는것과 관계가 있음을 시사하나 근력 운동을 활발히 한 노년층에서 유산소성 운동과 걷기 운동도 활발하게 할 수 있다는 점을 감안하여 결과를 해석할 필요가 있다. 본 연구 결과, 노년층에서는 근력 운동과 걷기 운동에서 매우 활동적인 그룹이 활동적인 그룹보다 더 많은 양상을 띄는 특징을 나타냈다. 이는 2020년 국민생활체육조사 결과 현대인이 신체활동을 하지 못하는 이유 1순위가 시간 부족임을 고려할 때, 노년층은 청년층이나 중년층에 비해 시간적 여유가 많기 때문에 근력 운동 또는 걷기 운동에 많은 시간을 할애할 수 있었음을 추정할 수 있다 [27]. 또한 각종 매체를 통해 노년층에서의 근력 운동 실천이 골다공증 및 치매와 같은 만성질환 예방과 관련하여 강조되고 있는 점과 걷기 운동이 신체에 무리가 덜 된다는 점이 노년층에서 두 운동의 실천이 매우 활동적인 수준으로 실천하게 되는 것과 연관성이 있음을 추정할 수 있다 [28, 29].

본 연구는 다음과 같은 한계를 가지고 있다. 첫째, 단면 연구로 인과관계를 규명할 수 없는 한계를 가지고 있다. 연령대에 맞는 신체활동 수준과 낮은 악력과의 인과관계를 구명하기 위한 전향적 코호트 연구(prospective cohort studies)가 필요할 것으로 생각된다. 둘째, 신체활동을 유산소성 운동, 근력 운동, 걷기로 구분하여 신체활동이 매우 활동적인 그룹과 활동적인 그룹, 비활동적인 그룹을 분류하였으나 각 신체활동 간 명확한 구분이 있는 것은 아니다. 본 연구는 해당 신체활동 수준이 낮은 악력에 미치는 관계를 보고자 한 것이며 신체활동 간 중복되는 연구 대상자를 제거할 경우 표본수가 급격히 줄어 한국인을 대표하는 데이터의 특성이 떨어질 수 있음을 고려했기 때문이다. 앞으로 신체활동 간 교차 효과를 고려 또는 신체활동을 명확히 구분한 추가적인 연구가 더 필요할 것이다. 셋째, 신체활동 실천에 관한 정보는 모두 자기 보고식 설문(self-reported questionnaire) 결과를 활용하여 분석되었기 때문에 신체활동과 낮은 악력의 연관성은 과대 또는 과소평가 됐을 가능성이 있다. 마지막으로 본 연구는 한국인을 대상으로 한 국민건강영양조사(KNHANES)의 데이터를 활용하여 다른 국가에 일반화하기에 어려운 한계가 있다. 이러한 제한점에도 불구하고 해당 연구는 한국 성인의 낮은 악력과 신체활동의 연관성에 대한 정보를 제공하여 노년층뿐만 아니라 젊은 층을 포함한 근감소증의 예방 전략 수립의 기초자료로 활용될 수 있을 것이다.

**CONFLICT OF INTEREST**

The authors have no conflicts of interest to declare for this study.

**FUNDING**

This study was financially supported by National Cancer Center (NCC-2010200).

**AUTHOR CONTRIBUTIONS**

Conceptualization: HSA, MK. Methodology, Formal analysis, Data curation, Writing- Original draft: HSA. Writing- Reviewing and Editing: HSA, HYC, MK. Funding acquisition: MK. Project administration: MK. Visualization: HAS, HYC.

**REFERENCES**

1. Cruz-Jentoft AJ, Bahat G, Bauer J, Boirie Y, Bruyere O, Cederholm T, et al. Sarcopenia: revised European consensus on definition and diagnosis. Age Ageing. 2019;48(1):16-31. Epub 2018/10/13. doi: 10.1093/ageing/afy169. PubMed PMID: 30312372; PubMed Central PMCID: PMCPMC6322506.

2. Anker SD, Morley JE, von Haehling S. Welcome to the ICD-10 code for sarcopenia. J Cachexia Sarcopenia Muscle. 2016;7(5):512-4. Epub 2016/11/29. doi: 10.1002/jcsm.12147. PubMed PMID: 27891296; PubMed Central PMCID: PMCPMC5114626.

3. Cruz-Jentoft AJ, Baeyens JP, Bauer JM, Boirie Y, Cederholm T, Landi F, et al. Sarcopenia: European consensus on definition and diagnosis: Report of the European Working Group on Sarcopenia in Older People. Age Ageing. 2010;39(4):412-23. Epub 20100413. doi: 10.1093/ageing/afq034. PubMed PMID: 20392703; PubMed Central PMCID: PMC2886201.

4. Beaudart C, Dawson A, Shaw SC, Harvey NC, Kanis JA, Binkley N, et al. Nutrition and physical activity in the prevention and treatment of sarcopenia: systematic review. Osteoporos Int. 2017;28(6):1817-33. Epub 2017/03/03. doi: 10.1007/s00198-017-3980-9. PubMed PMID: 28251287; PubMed Central PMCID: PMCPMC5457808.

5. Bosaeus I, Rothenberg E. Nutrition and physical activity for the prevention and treatment of age-related sarcopenia. Proc Nutr Soc. 2016;75(2):174-80. Epub 2015/12/02. doi: 10.1017/S002966511500422X. PubMed PMID: 26620911.

6. Marzetti E, Calvani R, Tosato M, Cesari M, Di Bari M, Cherubini A, et al. Physical activity and exercise as countermeasures to physical frailty and sarcopenia. Aging Clin Exp Res. 2017;29(1):35-42. Epub 2017/02/10. doi: 10.1007/s40520-016-0705-4. PubMed PMID: 28181204.

7. McPhee JS, French DP, Jackson D, Nazroo J, Pendleton N, Degens H. Physical activity in older age: perspectives for healthy ageing and frailty. Biogerontology. 2016;17(3):567-80. Epub 2016/03/05. doi: 10.1007/s10522-016-9641-0. PubMed PMID: 26936444; PubMed Central PMCID: PMCPMC4889622.

8. Foster C, Armstrong MEG. What types of physical activities are effective in developing muscle and bone strength and balance? J Frailty Sarcopenia Falls. 2018;3(2):58-65. Epub 2018/06/01. doi: 10.22540/JFSF-03-058. PubMed PMID: 32300694; PubMed Central PMCID: PMCPMC7155324.

9. Labott BK, Bucht H, Morat M, Morat T, Donath L. Effects of Exercise Training on Handgrip Strength in Older Adults: A Meta-Analytical Review. Gerontology. 2019;65(6):686-98. Epub 2019/09/10. doi: 10.1159/000501203. PubMed PMID: 31499496.

10. Lee YL, Lee BH, Lee SY. Handgrip Strength in the Korean Population: Normative Data and Cutoff Values. Annals of Geriatric Medicine and Research. 2019;23(4):183-9. doi: 10.4235/agmr.19.0042.

11. Kim CR, Jeon YJ, Jeong T. Risk factors associated with low handgrip strength in the older Korean population. PLoS One. 2019;14(3):e0214612. Epub 2019/03/29. doi: 10.1371/journal.pone.0214612. PubMed PMID: 30921399; PubMed Central PMCID: PMCPMC6438516.

12. Dodds R, Kuh D, Aihie Sayer A, Cooper R. Physical activity levels across adult life and grip strength in early old age: updating findings from a British birth cohort. Age Ageing. 2013;42(6):794-8. Epub 2013/08/29. doi: 10.1093/ageing/aft124. PubMed PMID: 23981980; PubMed Central PMCID: PMCPMC3809720.

13. Kim S-H, Lim B-O, An K-O. Association of Physical Activity and Handgrip Strength among Korean Elderly. The Asian Journal of Kinesiology. 2019;21(4):16-21. doi: 10.15758/ajk.2019.21.4.16.

14. Yoo JI, Choi H, Ha YC. Mean Hand Grip Strength and Cut-off Value for Sarcopenia in Korean Adults Using KNHANES VI. J Korean Med Sci. 2017;32(5):868-72. Epub 2017/04/06. doi: 10.3346/jkms.2017.32.5.868. PubMed PMID: 28378563; PubMed Central PMCID: PMCPMC5383622.

15. Lim SH, Kim YH, Lee JS. Normative Data on Grip Strength in a Population-Based Study with Adjusting Confounding Factors: Sixth Korea National Health and Nutrition Examination Survey (2014-2015). Int J Environ Res Public Health. 2019;16(12). Epub 2019/06/28. doi: 10.3390/ijerph16122235. PubMed PMID: 31242569; PubMed Central PMCID: PMCPMC6616518.

16. Global Physical Activity Questionnaire Analysis Guide (Version 2). 2005 [cited 2021 Mar 21]. Available from: https://www.who.int/ncds/surveillance/steps/resources/GPAQ_Analysis_Guide.pdf.

17. Gi YM, Jung B, Kim KW, Cho JH, Ha IH. Low handgrip strength is closely associated with anemia among adults: A cross-sectional study using Korea National Health and Nutrition Examination Survey (KNHANES). PLoS One. 2020;15(3):e0218058. Epub 2020/03/21. doi: 10.1371/journal.pone.0218058. PubMed PMID: 32196502; PubMed Central PMCID: PMCPMC7083305.

18. Su Y, Hirayama K, Han TF, Izutsu M, Yuki M. Sarcopenia Prevalence and Risk Factors among Japanese Community Dwelling Older Adults Living in a Snow-Covered City According to EWGSOP2. J Clin Med. 2019;8(3). Epub 2019/03/03. doi: 10.3390/jcm8030291. PubMed PMID: 30823497; PubMed Central PMCID: PMCPMC6463161.

19. Bae EJ, Kim YH. Factors Affecting Sarcopenia in Korean Adults by Age Groups. Osong Public Health Res Perspect. 2017;8(3):169-78. Epub 2017/08/07. doi: 10.24171/j.phrp.2017.8.3.03. PubMed PMID: 28781939; PubMed Central PMCID: PMCPMC5525561.

20. Al-Obaidi S, Al-Sayegh N, Nadar M. Smoking impact on grip strength and fatigue resistance: implications for exercise and hand therapy practice. J Phys Act Health. 2014;11(5):1025-31. Epub 2013/06/27. doi: 10.1123/jpah.2011-0357. PubMed PMID: 23799259.

21. Kim S, Choi S, Yoo J, Lee J. Association of Grip Strength with All-Cause Mortality and Cause-Specific Mortality: Analysis of the Korean Longitudinal Study of Ageing (2006–2016). Korean Journal of Family Practice. 2019;9(5):438-47. doi: 10.21215/kjfp.2019.9.5.438.

22. Lee EJ, Yang SK, Lee DC. Smoking and its Relationship with Sarcopenia: Result from the 2008-2011 Korean National Health and Nutrition Examination Survey. Korean Journal of Family Practice. 2018;8(5):752-8. doi: 10.21215/kjfp.2018.8.5.752.

23. Pang BWJ, Wee SL, Lau LK, Jabbar KA, Seah WT, Ng DHM, et al. Prevalence and Associated Factors of Sarcopenia in Singaporean Adults-The Yishun Study. J Am Med Dir Assoc. 2020. Epub 2020/07/23. doi: 10.1016/j.jamda.2020.05.029. PubMed PMID: 32693999.

24. Gaba A, Cuberek R, Svoboda Z, Chmelik F, Pelclova J, Lehnert M, et al. The effect of brisk walking on postural stability, bone mineral density, body weight and composition in women over 50 years with a sedentary occupation: a randomized controlled trial. BMC Womens Health. 2016;16(1):63. Epub 20160921. doi: 10.1186/s12905-016-0343-1. PubMed PMID: 27653632; PubMed Central PMCID: PMC5031336.

25. Janssen I, Heymsfield SB, Wang Z, et al. Skeletal muscle mass and distribution in 468 men and women aged 18–88 yr. J Appl Physiol 2000;89:81–8. doi: 10.1152/jappl.2000.89.1.81. PubMed PMID: 10904038

26. Seong JY, Ahn HY, Park Y, Shin S, Ha IH. Association Between Aerobic Exercise and Handgrip Strength in Adults: A Cross-Sectional Study Based on Data from the Korean National Health and Nutrition Examination Survey (2014-2017). J Nutr Health Aging. 2020;24(6):619-26. Epub 2020/06/09. doi: 10.1007/s12603-020-1372-x. PubMed PMID: 32510115.

27. Ministry of Culture, Sports and Tourism. National sport participation survey 2020 [cited 2021 Dec]. Available from: https://www.mcst.go.kr/attachFiles/viewer/skin/doc.html?fn= DEPTDATA_20201230092110031898.pdf&rs = /attachFiles/viewer/result/202204/ (Korean).

28. Pinheiro MB, Oliveira J, Bauman A, Fairhall N, Kwok W, Sherrington C. Evidence on physical activity and osteoporosis prevention for people aged 65+ years: a systematic review to inform the WHO guidelines on physical activity and sedentary behaviour. Int J Behav Nutr Phys Act. 2020;17(1):150. Epub 2020/11/27. doi: 10.1186/s12966-020-01040-4. PubMed PMID: 33239014; PubMed Central PMCID: PMCPMC7690138.

29. Lee J. Effects of Aerobic and Resistance Exercise Interventions on Cognitive and Physiologic Adaptations for Older Adults with Mild Cognitive Impairment: A Systematic Review and Meta-Analysis of Randomized Control Trials. Int J Environ Res Public Health. 2020;17(24). Epub 2020/12/16. doi: 10.3390/ijerph17249216. PubMed PMID: 33317169; PubMed Central PMCID: PMCPMC7764103.
